# Nicotinamide Riboside-Conditioned Microbiota Deflects High-Fat Diet-Induced Weight Gain in Mice

**DOI:** 10.1128/msystems.00230-21

**Published:** 2022-01-25

**Authors:** Valery V. Lozada-Fernández, Orlando deLeon, Stephanie L. Kellogg, Fatima L. Saravia, Matthew A. Hadiono, Samantha N. Atkinson, Justin L. Grobe, John R. Kirby

**Affiliations:** a Department of Microbiology and Immunology, Medical College of Wisconsin, Milwaukee, Wisconsin, USA; b Department of Pediatrics, Medical College of Wisconsin, Milwaukee, Wisconsin, USA; c Department of Physiology, Medical College of Wisconsin, Milwaukee, Wisconsin, USA; d Department of Medicine, University of Chicago, Chicago, Illinois, USA; Johns Hopkins Bloomberg School of Public Health

**Keywords:** diet-induced obesity, energy expenditure, fecal material transfer, high-fat diet, nicotinamide riboside

## Abstract

The gut microbiome plays an essential role in host energy homeostasis and influences the development of obesity and related conditions. Studies demonstrate that nicotinamide riboside (NR) supplementation for diet-induced obesity (DIO) reduces weight gain and increases energy expenditure in mice. NR is a vitamin B_3_ derivative and an NAD^+^ precursor with potential for treating human diseases arising from mitochondrial degeneration, including obesity and type 2 diabetes. Gut bacteria produce vitamin B_3_ in the colon and are capable of salvaging and metabolizing vitamin B_3_ and its derivatives. However, it is unknown how dietary supplementation of NR alters the microbiome and if those alterations contribute to deflection of weight gain. In this study, we fed C57BL/6J male mice a high-fat diet (HFD) supplemented with or without NR and performed a fecal material transfer (FMT) to establish a link between NR-conditioned microbiota and NR-induced deflection of weight gain. FMT from NR-treated donors to naive mice fed a HFD was sufficient to deflect weight gain by increasing energy expenditure. We also investigated the effects of NR on the microbiome by using metagenomics sequencing. We found that NR-treated mice displayed an altered gut microbial composition relative to controls and that fecal transplant resulted in a distinct functional metabolic profile characterized by enrichment of butyrate-producing *Firmicutes*. NR-treated donors and subsequent FMT recipients share a similar enrichment of metagenomic biomarkers relative to controls. These findings suggest that microbial factors contribute to the beneficial effects of dietary NR supplementation, which may be useful to enhance the therapeutic effects of NR.

**IMPORTANCE** With obesity and type 2 diabetes (T2D) at epidemic levels, we need to understand the complex nature of these diseases to design better therapeutics. The underlying causes of both obesity and T2D are complex, but both are thought to develop, in part, based on contributions from the gut microbiota. Nicotinamide riboside is a gut-derived vitamin B_3_ derivative and NAD^+^ precursor which has the potential to treat and prevent metabolic disorders by ameliorating mitochondrial dysfunction. Understanding how NR affects the gut microbiome and whether NR-conditioned microbiota contributes to weight loss in the host would (i) improve diagnosis and treatments for obesity and other metabolic pathologies, (ii) tailor treatments to satisfy the needs of each individual moving toward the future of precision medicine, and (iii) benefit other scientific fields that currently investigate the effects of NR in other disease pathologies.

## INTRODUCTION

In 2016, the Centers for Disease Control and Prevention (CDC) reported that the prevalence of obesity in the United States was 39.8% in adults and 18.5% in children (1). Obesity is defined as a body mass index (BMI) of 30 or more (2) and results from an energy imbalance leading to excessive accumulation of fat (1, 3). This disease arises through a combination of genetic and environmental factors, including dietary interventions and sedentary lifestyles, and is still not well understood. People who are obese often develop comorbidities such as type 2 diabetes (T2D), cancer, hypertension, and cardiovascular diseases (4). Currently available treatments for obesity include significant lifestyle changes, gastric bypass surgery, and the use of dietary supplements. However, these interventions have not decelerated the obesity epidemic, and thus, there is an urgent need to develop new antiobesity therapies.

The gut microbiome is a metabolic organ (5) because it aids the host in many processes that modulate host physiology and metabolism (6), such as digestion, vitamin and specialized metabolite production, and xenobiotic detoxification. Subsequently, alterations to taxonomic composition and functional profiles of the gut microbiota are linked to metabolic syndrome and associated disorders, including obesity (7–9). Decreased microbial gene diversity is observed in patients with obesity in comparison with lean patients (10). Furthermore, a fecal material transfer (FMT) from obese to germfree mice is sufficient to induce an obesity phenotype in the recipient (9, 11, 12). Therefore, bacteria play a direct role in the development of obesity in the host.

Nicotinamide riboside (NR) is a vitamin B_3_ derivative and NAD^+^ precursor that has been the subject of recent studies and clinical trials as a potential treatment for human diseases that arise from mitochondrial degeneration, such as obesity and T2D (13). NR is found in our diet (13, 14) and can be synthesized by the gut microbiota. In a mouse model of diet-induced obesity, male mice fed a 60% high-fat diet (HFD) supplemented with NR had a reduction in diet-induced weight gain due to increased energy expenditure and had improved obesity-related conditions (15). A mechanistic model was proposed in which NR supplementation increases NAD^+^ in key metabolic tissues, activating a class of NAD-dependent deacetylases known as sirtuins, which further modulate key metabolic regulators to increase energy expenditure (15, 16). However, there remains the likelihood of additional underlying mechanisms by which NR could alter host metabolism and physiology.

Some bacteria produce and modify B vitamins and their derivatives in the colon. As an example, from the suggested 15 mg/day of niacin (nicotinamide and nicotinic acid), 27% (4.05 mg/day niacin) is predicted to be synthesized by gut microbes (17). Additionally, some gut bacteria can salvage vitamin B_3_ derivatives such as NR using dedicated transporters for NAD^+^ synthesis (13, 18). Because some gut bacteria are capable of importing and metabolizing NR, we hypothesize that dietary NR supplementation alters the gut microbiome to subsequently mediate some of the benefits seen in the host.

To test this hypothesis, we fed male C57BL/6J mice a 60% HFD supplemented with or without NR and then performed a fecal material transfer into naive recipients. Throughout the experiment, we monitored weight gain, collected fecal samples for microbiome analysis, and assessed energy balance. We found that dietary NR supplementation alters the gut microbiome compositional and functional profiles, corresponding to a reduction in HFD-induced weight gain and decreased energy efficiency, relative to controls. Transfer of fecal material from NR-treated donors into naive recipients was sufficient to reduce HFD-induced weight gain, decrease energy efficiency, and increase the relative abundance of *Firmicutes* predicted to be butyrate producers. We conclude that NR-conditioned microbiota are capable of affecting the overall metabolic status of the host.

## RESULTS

### NR supplementation reduces weight gain caused by a HFD.

**FIG 1:**
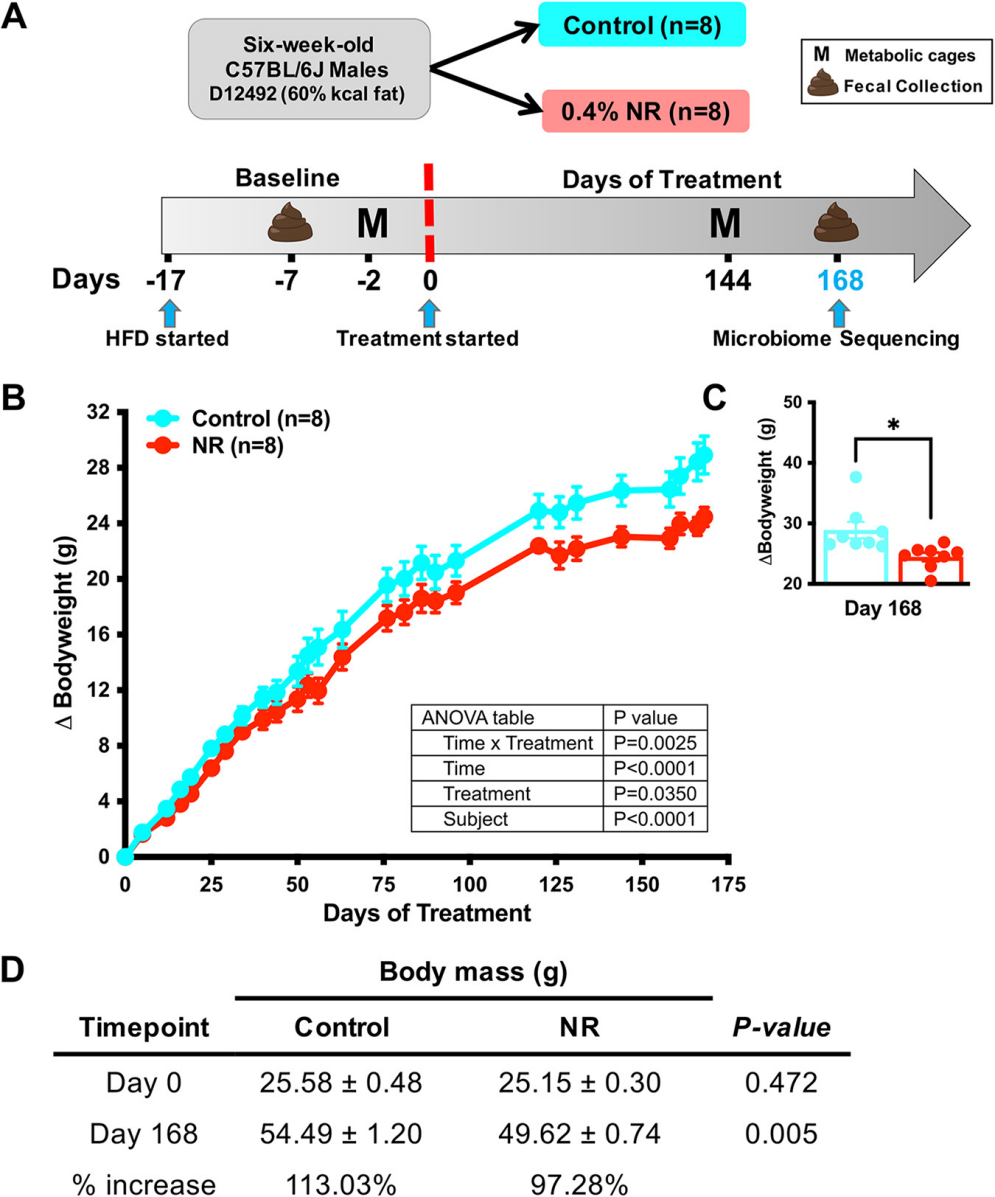
Dietary NR supplementation deflects HFD-induced weight gain. (A) Six-week-old male C57BL/6J mice were placed on a high-fat diet (HFD) when they arrived and were on a baseline period for 2 weeks. Mice were weight matched, randomized, and divided into groups. NR-treated mice were supplemented with 0.4% NR, whereas for the control group, the diet was mixed with water (vehicle). Mice were housed in metabolic cages (M) during baseline and at the end of the experiment. Feces were collected for shotgun metagenomic sequencing at day 168. (B) NR-treated mice (red, *n* = 8) exhibited a statistically significant reduction in weight gain compared to control mice (cyan, *n* = 8). Data were analyzed by two-way ANOVA (*, *P* < 0.05). (C) After 168 days of NR treatment, there was a weight gain difference of 4.9 g between treatment groups. Data were analyzed by an unpaired *t* test with Welch’s correction (*, *P* < 0.05). (D) At day 0, no statistically significant differences in body mass were found between groups, but after 168 days of treatment, there was a significant reduction in body mass in NR-treaded mice relative to control mice. Data were analyzed by an unpaired *t* test with Welch’s correction (*, *P* < 0.05). All data are represented as the mean ± SEM.

Nicotinamide riboside (NR) is known to deflect weight gain in response to a high-fat diet (HFD) in mice (15). However, the mechanisms affecting weight gain in response to NR remain unknown. Because gut bacteria have been implicated in affecting body mass in multiple studies (8, 12, 19–24), we hypothesized that gut flora is partially responsible for the response to NR in HFD mice. To test this, we fed male C57BL/6J mice a 60% HFD supplemented with 0.4% NR or vehicle (water), measured weight gain (Fig. 1A), and collected fecal samples for both sequencing and subsequent transfers (see below). Supplementation with 0.4% NR was chosen based on previous studies demonstrating beneficial metabolic effects (15, 25–27). After 144 days, the increase in body mass for NR-treated mice was significantly less than that of controls and continued to the end of the experiment (Fig. 1B). After 168 days of treatment, there was a 4.9 g difference between the groups (Fig. 1C; *, *P* < 0.05). At day 0, there was no statistically significant difference in body mass between groups. After 168 days of NR treatment, there was a significant reduction in body mass compared to that of the control, which resulted in a 113.03% and 97.28% increase in body mass in control and NR-treated mice, respectively (Fig. 1D; *, *P* < 0.05). The observed 15.75% reduction in weight gain for NR-treated mice was accompanied by a reduction in fasting blood glucose levels (see Fig. S1 in the supplemental material).

10.1128/mSystems.00230-21.1 FIG S1 NR treatment, but not FMT-NR, reduces fasting blood glucose levels. Mice were fasted for hours. Blood was drawn from the tail vein, and glucose was measured using a glucometer. (A) NR-treated mice exhibited lower fasting blood glucose levels than control mice. (B) In contrast, no significant differences in fasting blood glucose levels were found between FMT-NR and FMT-control mice. Data are represented as the mean ± SEM. Data were analyzed by two-tailed Student's *t* test. *, *P <* 0.05. Download FIG S1, PDF file, 0.1 MB. Copyright © 2022 Lozada-Fernández et al. 2022 Lozada-Fernández et al. https://creativecommons.org/licenses/by/4.0/ This content is distributed under the terms of the Creative Commons Attribution 4.0 International license.

### NR supplementation alters the gut microbiota.

**FIG 2:**
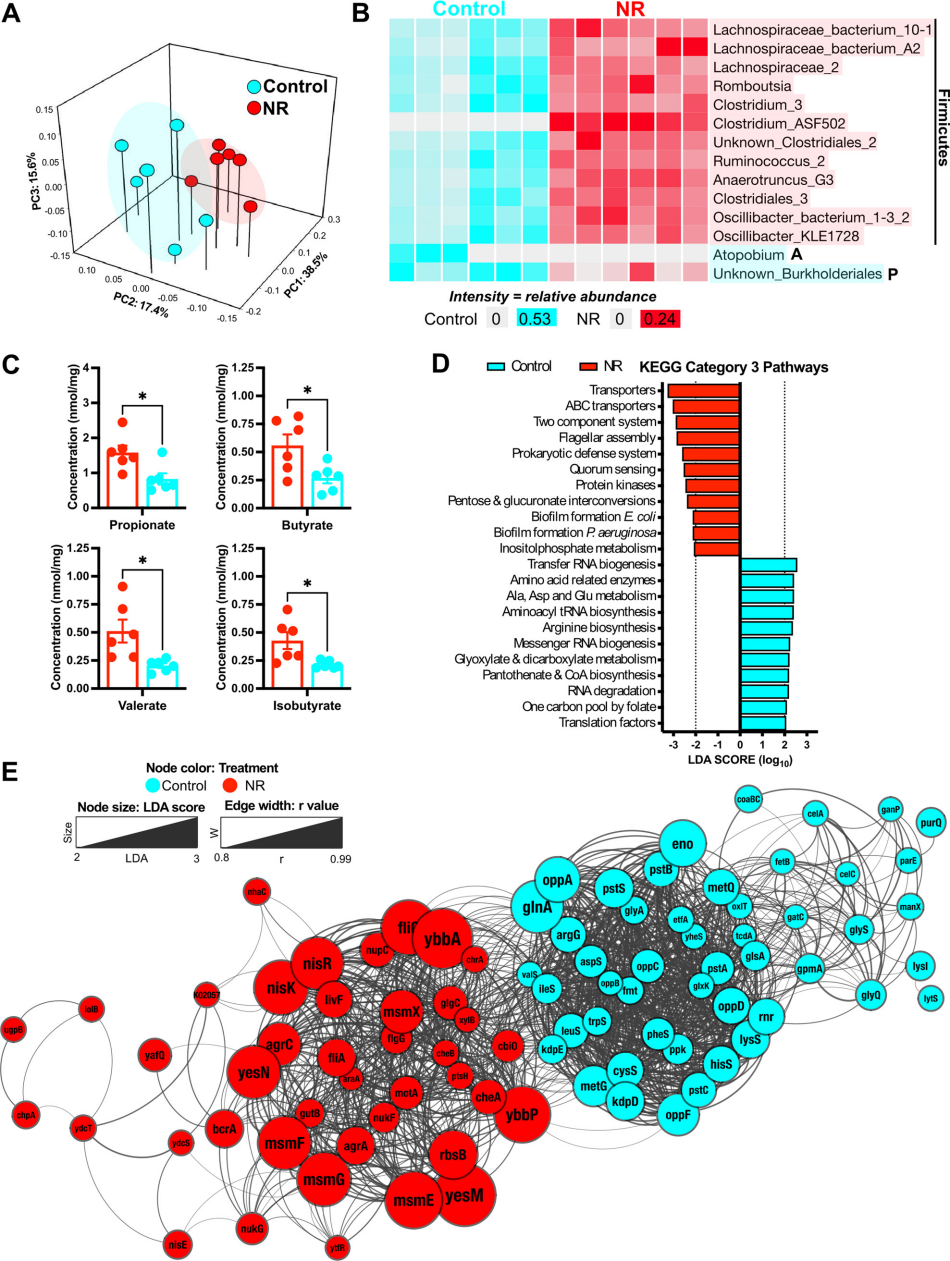
NR supplementation alters the gut microbiota compositional and functional profiles. To study the gut microbiota composition, feces from NR-treated (*n* = 6) and control (*n* = 6) mice were collected and subjected to shotgun metagenomic sequencing. (A) Principal-coordinate analysis (PCoA) plot of Bray-Curtis distances illustrating differences in the gut microbiome between treatment groups at day 168. By implementing shotgun metagenomic sequencing, we found that NR-treated mice (red) exhibited a unique gut microbiome (PERMANOVA; *, *P* < 0.05) compared to control mice (cyan). (B) Anvi’o plot illustrating the relative abundance of 14 discriminatory MAGs identified by linear discriminant analysis effect size (LEfSe). The LEfSe analysis was performed using all 88 MAGs. MAGs are ordered by phylogenetic tree. Taxon names highlighted in cyan represent MAGs enriched in control mice, whereas the taxon names highlighted in red were found enriched in NR mice. The majority of MAGs belong to the *Firmicutes* phylum, whereas the MAGs Unknown_Burkholderiales and Atopobium belong to *Proteobacteria* and *Actinobacteria*, respectively. (C) Fecal SCFA concentrations. NR-treated mice exhibited a significant increase in fecal propionate, butyrate, valerate, and isobutyrate compared to control mice. All values were normalized by milligram of feces. Data were analyzed by an unpaired *t* test with Welch’s correction (*, *P* < 0.05). All data are represented as the mean ± SEM. (D) Discriminant KEGG pathways enriched in NR-treated mice and control mice. The Q2Q3 coverage (defined by anvi’o as the average depth of coverage excluding nucleotide positions with coverages in the 1st and 4th quartiles) of KEGG pathways was analyzed using LEfSe. The significant LDA scores are colored cyan for the control group, whereas the scores for the NR-treated mice are colored red. (E) Network visualization of discriminant KOs enriched in NR-treated and control mice. We performed LEfSe on all KOs from enriched pathways and found 90 discriminant KOs. The network was visualized and annotated in Cytoscape. Node size represents LDA scores from discriminant KOs, whereas the edge width represents *r* values calculated from Pearson correlations (0.8 cutoff). The compound spring embedded (CoSE) layout was applied to the network.

To assess changes in the gut microbiota functional capacity in response to NR treatment, we extracted DNA from fecal samples of NR-treated and control mice (*n* = 6) and subjected it to paired-end shotgun metagenomic sequencing using the NovaSeq Illumina platform (see Materials and Methods). Eighty-eight metagenome-assembled genomes (MAGs) were generated using CONCOCT, followed by manual refinement (Table S1). Based on available samples from all cohorts on day 168, we found that NR-treated mice exhibited a unique gut microbiota composition in comparison to controls (Fig. 2A; permutational multivariate analysis of variance [PERMANOVA], *, *P* < 0.05).

10.1128/mSystems.00230-21.5 TABLE S1 Summary table of MAGs generated from the dietary NR supplementation experiment. Bins were generated by CONCOCT and then manually refined to satisfy a completion of at least 50% or 2 Mbp and a redundancy of no more than 10%. Download Table S1, PDF file, 0.1 MB. Copyright © 2022 Lozada-Fernández et al. 2022 Lozada-Fernández et al. https://creativecommons.org/licenses/by/4.0/ This content is distributed under the terms of the Creative Commons Attribution 4.0 International license.

To further discern the microbiome taxonomical differences, we used the linear discriminant analysis (LDA) effect size (LEfSe) algorithm to determine discriminatory MAGs enriched in each treatment group. Two MAGs belonging to the phyla *Proteobacteria* (P) and *Actinobacteria* (A) were enriched in control-treated mice (Unknown_Burkholderiales and Atopobium, respectively), whereas the NR-treated mice exhibited enrichment of 12 MAGs, all belonging to the *Firmicutes* (Fig. 2B; Fig. S2).

10.1128/mSystems.00230-21.2 FIG S2 (A) Relative abundance of MAGs generated from the dietary supplementation experiment. The bar graph at the bottom shows the relative abundance of all 88 MAGs collapsed to the phylum. The *Firmicutes* phylum was divided into two groups: the enriched and not-enriched *Firmicutes*. The “Firmicutes enriched” group contains the 12 MAGs found enriched via LEfSe, whereas the “Firmicutes not enriched” group consists of the other 66 MAGs. Overall, NR-treated mice exhibited a higher relative abundance of *Firmicutes*, in which the “Firmicutes enriched” group represents approximately 17% of the total community. (B) Relative abundance of MAGs generated from the FMT experiment. The bar graph at the bottom shows the relative abundance of all 78 MAGs collapsed to the phylum. The *Firmicutes* phylum was divided into two groups: the “Firmicutes enriched” and “Firmicutes not enriched.” The “Firmicutes enriched” group contains the 20 MAGs found enriched via LEfSe, whereas the “Firmicutes not enriched” group consists of the other 44 MAGs. Overall, FMT-NR-treated mice exhibited a higher relative abundance of *Firmicutes*, in which the “Firmicutes enriched” group represents approximately 30% of the total community. Download FIG S2, PDF file, 0.3 MB. Copyright © 2022 Lozada-Fernández et al. 2022 Lozada-Fernández et al. https://creativecommons.org/licenses/by/4.0/ This content is distributed under the terms of the Creative Commons Attribution 4.0 International license.

*Firmicutes* are major butyrate producers in the gut, whereas members of the *Proteobacteria* are known to oxidize butyrate for growth (28, 29). This led to the hypothesis that dietary NR supplementation enriches for *Firmicutes* capable of butyrate synthesis. To test this, we searched for KEGG orthology genes (KOs) involved in butyrate biosynthesis pathways in the enriched MAGs of each treatment group (Fig. S3), and we found a total of 27 KOs. The enriched *Firmicutes* MAGs within the NR-treated group included KOs from the acetyl coenzyme A (acetyl-CoA) butyrate synthesis pathway (Fig. S3). For the MAGs enriched in control mice, we identified only the *hbd* gene from the acetyl-CoA pathway and the genes *etfA* and *etfB*, which catalyze the conversion of crotonoyl-CoA into butyryl-CoA, a common step among the four known butyrate synthesis pathways (29).

10.1128/mSystems.00230-21.3 FIG S3 Normalized coverage of genes in the acetyl-CoA pathway for butyrate synthesis within the 12 enriched MAGs. (A) Dietary experiment. The acetyl-CoA pathway for butyrate synthesis was the most common pathway found within the 12 enriched *Firmicutes* MAGs in the NR-treated samples. Only one gene was found within the enriched *Actinobacteria* member, whereas no KOs were found in the enriched *Proteobacteria*. (B) FMT experiment. The acetyl-CoA pathway for butyrate synthesis was also the most common pathway found within the 19 enriched *Firmicutes* MAGs in the FMT-NR-treated mice. Only two genes were found within the enriched *Proteobacteria* member. Download FIG S3, PDF file, 0.2 MB. Copyright © 2022 Lozada-Fernández et al. 2022 Lozada-Fernández et al. https://creativecommons.org/licenses/by/4.0/ This content is distributed under the terms of the Creative Commons Attribution 4.0 International license.

Based on the observed enrichment for butyrate biosynthetic genes, we predicted that stool might contain elevated butyrate in NR-treated animals. Thus, we conducted liquid chromatography-mass spectrometry (LC-MS) metabolomics profiling to determine short-chain fatty acid (SCFA) levels in stool. We found a significant increase in fecal propionate, butyrate, valerate, and isobutyrate in NR-treated mice compared to that in controls (Fig. 2C), whereas no significant differences between treatment groups were found in fecal acetate, isovalerate, caproate, and heptanoate (Fig. S4). Together, these results indicate that dietary NR supplementation enriches for butyrate-producing *Firmicutes*.

10.1128/mSystems.00230-21.4 FIG S4 Measurement of SCFA concentration. Fecal acetate (A), isovalerate (B), caproate (C), and heptanoate (D) concentrations in the dietary mouse cohort. Fecal acetate (E), isovalerate (F), caproate (G), and heptanoate (H) concentrations in the FMT mouse cohort. All values were normalized by milligram of feces. Download FIG S4, PDF file, 0.1 MB. Copyright © 2022 Lozada-Fernández et al. 2022 Lozada-Fernández et al. https://creativecommons.org/licenses/by/4.0/ This content is distributed under the terms of the Creative Commons Attribution 4.0 International license.

Considering that NR-treated mice exhibited significant compositional differences regarding butyrate biosynthetic genes and composition, we searched for additional functional differences by implementing the LEfSe algorithm at the category 3 pathway level (Fig. 2D). We found 11 pathways enriched in NR-treated MAGs, including ABC transporters, two-component systems, transporters, flagellar assembly, prokaryotic defense systems, quorum sensing, pentose and glucuronate interconversions, protein kinases, inositol phosphate metabolism, and biofilm formation pathways also found in Escherichia coli and Pseudomonas aeruginosa. In control mice, we found 11 pathways enriched, including amino acid-related enzymes, alanine, aspartate, and glutamate metabolism, arginine biosynthesis, aminoacyl tRNA biosynthesis, tRNA biogenesis, mRNA biogenesis, glyoxylate and dicarboxylate metabolism, pantothenate and CoA biosynthesis, RNA degradation, translation factors, and one carbon pool by folate.

Given that NR supplementation causes major functional alterations in the murine gut microbiome, we investigated discriminant features by implementing LEfSe on the KOs from the enriched pathways (Fig. 2D). We identified 90 discriminant genes, which were then used to generate a distance matrix and gene network. We used Cytoscape to visualize, annotate, and apply the compound spring embedded (CoSE) layout. We found higher correlation values between KOs enriched within the same treatment group. In this manner, we found 41 enriched KOs in the NR-treated group and 49 discriminant KOs in the control group (Fig. 2E). These results further illustrate that dietary NR supplementation alter the murine gut microbiome compositional and functional profiles.

### NR-conditioned FMT reduces weight gain caused by a HFD.

**FIG 3:**
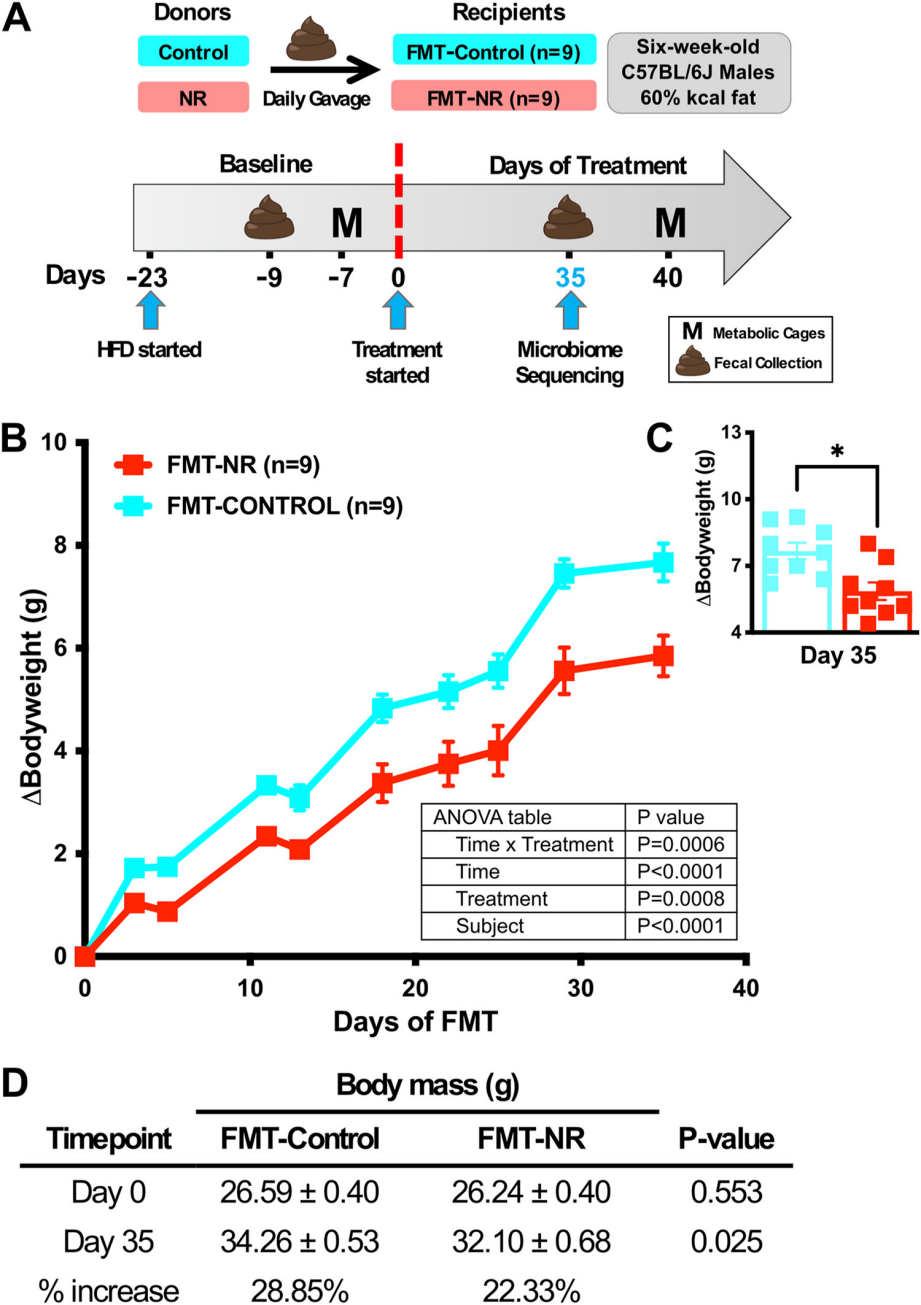
NR-conditioned fecal material transfer reduces HFD-induced weight gain. (A) Six-week-old male C57BL/6J mice fed a 60% HFD were on a baseline period for 3 weeks. Mice were weight matched, randomized, and divided into groups. Mice received 0.1 g feces/mL PBS from either NR-treated or control donors daily via oral gavage. Mice were housed in metabolic cages (M) during baseline and at the end of the experiment. Feces were collected for shotgun metagenomic sequencing at day 35. (B) Naive mice treated daily with feces from NR-treated donors (FMT-NR, red, *n* = 9) exhibited a reduction in weight gain relative to naive mice receiving feces from control donors (FMT-control, cyan, *n* = 9). Data were analyzed by two-way ANOVA (*, *P* < 0.05). (C) At day 35, there was a weight gain difference of 2.2 g between groups. Data were analyzed by an unpaired *t* test with Welch’s correction (*, *P* < 0.05). (D) At day 0, no statistically significant differences in body mass were found between groups. After 35 days of FMT-NR treatment, there was a significant reduction in body mass relative to that of the control. Data were analyzed by an unpaired *t* test with Welch’s correction (*, *P* < 0.05). All data are represented as the mean ± SEM.

Butyrate producers are known to decrease weight gain when used as a supplement in a diet-induced obesity (DIO) mouse model (30). Furthermore, because NR treatment increases energy expenditure (15, 16) and leads to an enrichment of butyrate producers, we hypothesized that the NR-conditioned microbiota contributes to the amelioration of high-fat diet (HFD)-induced weight gain. To test this hypothesis, we performed a fecal material transfer (FMT) from control or NR-treated donors into a cohort of naive recipients fed the same 60% HFD (Fig. 3A). An FMT from NR-treated mice into naive recipients (FMT-NR) was sufficient to reduce the HFD-induced weight gain (Fig. 3B). In contrast to dietary supplementation, a significant body weight difference of 2.2 g was observed at 35 days following the FMT from NR donor mice relative to controls (Fig. 3C). Mice receiving NR-conditioned FMT had a 22.33% increase in body mass compared to a 28.85% increase for the FMT-control group (Fig. 3D). These results indicate that the microbiota from NR-treated mice is sufficient to reduce HFD-induced weight gain by 6.52%. It is worth noting that in contrast to NR supplementation, the FMT from NR-treated mice did not influence fasting blood glucose levels (Fig. S1).

### NR-conditioned FMT remodels the gut microbiome similarly to NR supplementation.

**FIG 4:**
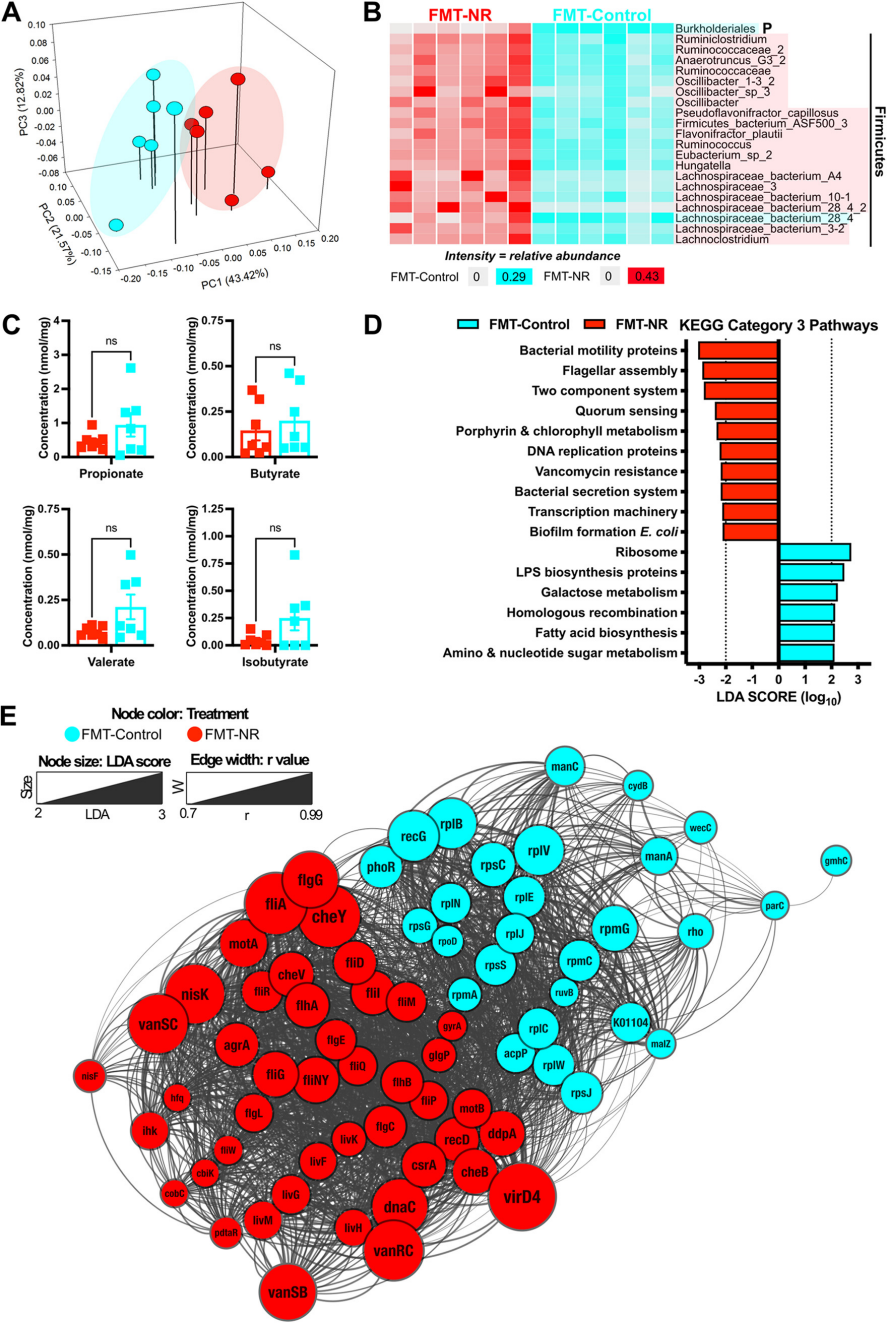
NR-conditioned fecal material transfer remodels the gut microbiome similarly to NR supplementation. (A) Principal-coordinate analysis (PCoA) plot of Bray-Curtis distances illustrating differences in the gut microbiome at day 35 from shotgun metagenomic sequencing. By implementing shotgun metagenomic sequencing, we found that FMT-NR-treated mice (red) exhibited a unique gut microbiome (PERMANOVA; *, *P* < 0.05) in comparison to FMT-control mice (cyan). (B) Anvi’o plot illustrating the relative abundance of 21 discriminatory MAGs identified by LEfSe. The analysis was performed using all 78 MAGs. MAGs are ordered by phylogenetic tree. Taxon names highlighted in cyan represent MAGs enriched in FMT-control mice, whereas the taxon names highlighted in red were found enriched in FMT-NR mice. The majority of MAGs belong to the *Firmicutes* phylum, whereas the Burkholderiales MAG belongs to the *Proteobacteria* phylum. (C) Fecal SCFA concentrations. No significant differences between FMT-NR-treated and FMT-control mice were found in fecal propionate, butyrate, valerate, and isobutyrate. All values were normalized by milligram of feces. Data were analyzed by an unpaired *t* test with Welch’s correction (*, *P* < 0.05; ns, nonsignificant). All data are represented as the mean ± SEM. (D) Discriminant KEGG pathways enriched in FMT-NR-treated mice and FMT-control mice. The Q2Q3 coverage of KEGG pathways was analyzed by LEfSe. The significant LDA scores are shown in cyan for pathways enriched in the FMT-control mice, whereas the pathways enriched in FMT-NR mice are shown red. (E) Network visualization of KOs enriched in FMT-NR-treated and FMT-control mice. We performed LEfSe on all KOs from discriminant pathways and found 72 discriminant KOs. The network was visualized and annotated in Cytoscape. The node size represents LDA scores from discriminant KOs, whereas the edge width represents *r* values calculated from Pearson correlations (0.7 cutoff). The compound spring embedded (CoSE) layout was applied to the network.

Because NR supplementation altered the gut microbiota and the FMT was effective in ameliorating weight gain, we assessed the microbiome after 35 days of FMT. By following the same approach described for the dietary supplementation, we performed shotgun metagenomic sequencing to assess the overall functional capacity of the resulting microbiota. We used the Illumina NovaSeq technology with paired-ended reads and a target of 10 million reads. Seventy-eight MAGs were generated using CONCOCT, followed by manual refinement (Table S2). We found that mice receiving the FMT from NR-treated donors (FMT-NR) exhibited a unique gut microbiota composition compared to mice receiving the FMT from control donors (FMT-control) at day 35 (Fig. 4A; PERMANOVA, *, *P* < 0.05).

10.1128/mSystems.00230-21.6 TABLE S2 Summary table of MAGs generated from the FMT experiment. Bins were generated by CONCOCT and then manually refined to satisfy a completion of at least 50% or 2 Mbp and a redundancy of no more than 10%. Download Table S2, PDF file, 0.1 MB. Copyright © 2022 Lozada-Fernández et al. 2022 Lozada-Fernández et al. https://creativecommons.org/licenses/by/4.0/ This content is distributed under the terms of the Creative Commons Attribution 4.0 International license.

To investigate taxonomical differences between treatment groups, we implemented LEfSe and identified 21 discriminatory MAGs (Fig. 4B and Fig. S2). The MAGs Burkholderiales and Lachnospiraceae_bacterium_28_4 were enriched in FMT-control mice, which belong to the *Proteobacteria* and *Firmicutes* phyla, respectively. For the FMT-NR mice, we found 19 discriminatory MAGs all belonging to *Firmicutes*, with various predicted butyrate producers. These results suggest that as with dietary NR supplementation, FMT-NR treatment enriches for members of the *Firmicutes*, with butyrate production capability.

By following the same analysis workflow as with the dietary NR supplementation, we searched for KOs in butyrate-producing pathways within the enriched MAGs. Overall, we found a total of 33 KOs that are part of the butanoate metabolism pathway. The most common butanoate KOs found within the enriched *Firmicutes* MAGs belong to the acetyl-CoA pathway (Fig. S3). For the *Proteobacteria* MAG, we found only the genes *etfA* and *etfB*, which convert crotonoyl-CoA into butyryl-CoA.

As described above, we conducted LC-MS metabolomics profiling to determine SCFA levels in stool following fecal material transfer from donors. In contrast to the observations for NR-supplemented mouse stool samples which were used as donor material, no significant differences for the recipients were identified for fecal SCFAs, including propionate, butyrate, valerate, and isobutyrate (compare Fig. 2C and Fig. 4C), nor for acetate, isovalerate, caproate, and heptanoate (Fig. S4). Together, these results suggest that daily FMT from NR-treated donors results in enrichment of genes for butyrate production in *Firmicutes*, but there were no significant differences in SCFA levels.

Considering that daily FMT-NR treatment caused taxonomical differences, we hypothesized that the FMT-NR mice would also exhibit functional differences compared to FMT-controls. To test this hypothesis, we performed a functional analysis at the category 3 pathway level using the LEfSe algorithm. We found 10 pathways enriched in FMT-NR mice, including bacterial motility, flagellar assembly, two-component systems, quorum sensing, biofilm formation, vancomycin resistance, porphyrin and chlorophyll metabolism, DNA replication proteins, transcription machinery, and bacterial secretion systems (Fig. 4D). For the FMT-control, we found six pathways enriched, including ribosome production, homologous recombination, lipopolysaccharide (LPS) biosynthesis, galactose metabolism, fatty acid biosynthesis, and amino sugar and nucleotide sugar metabolism (Fig. 4D). These results suggest that daily FMT-NR treatment alters the functional profile of the murine gut microbiome in comparison to that of controls.

Lastly, since FMT-NR supplementation causes major functional alterations in the murine gut microbiome, we investigated discriminant features by implementing LEfSe with the KOs from the enriched pathways (Fig. 4D). We identified 72 discriminant genes, which were then used to generate a distance matrix and gene network. Furthermore, we used Cytoscape to visualize, annotate, and apply the compound spring embedded (CoSE) layout. Similar to the results shown in Fig. 2E, we found higher correlation values between KOs enriched within the same treatment group. Specifically, we found 28 discriminant genes in the FMT-control mice and 44 discriminant genes in FMT-NR mice (Fig. 4E). These results further demonstrate that daily FMT-NR treatment alters the murine gut microbiome compositional and functional profiles.

### NR supplementation decreases energy efficiency in mice fed a high-fat diet.

**FIG 5:**
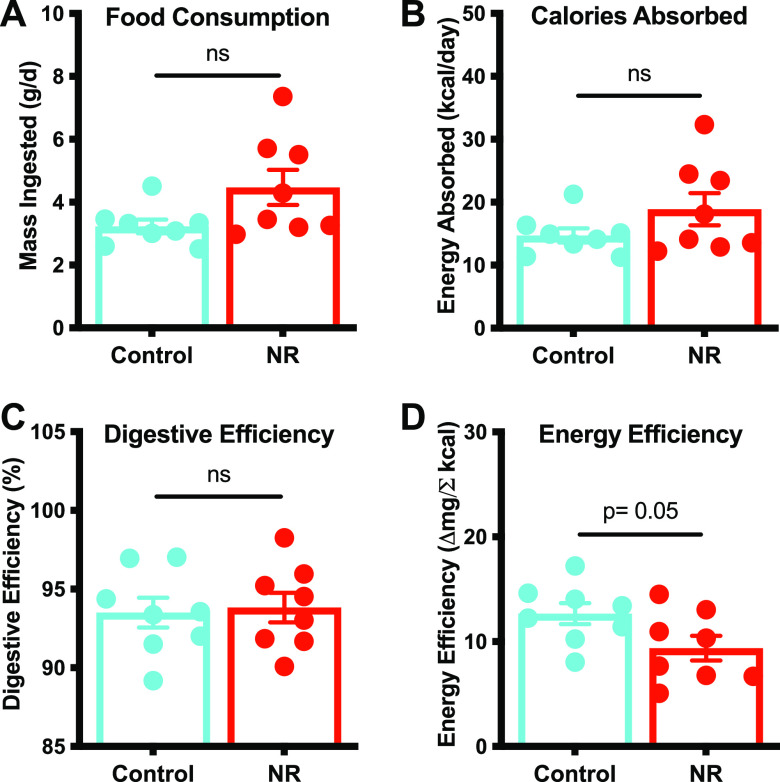
NR supplementation decreases energy efficiency in HFD-fed mice. To understand the underlying mechanism of weight gain, mice were housed in metabolic cages to quantitatively assess food and water intake and urine and fecal output. Feces were subjected to bomb calorimetry. No significant differences between treatment groups were found in food consumption (A), energy absorbed (B), or digestive efficiency (C). (D) NR-treated mice exhibited a decrease in energy efficiency compared to control mice (*P* value = 0.05). All data are represented as the mean ± SEM. Data were analyzed by an unpaired *t* test with Welch’s correction (*, *P* < 0.05).

Studies have shown that NR treatment significantly increases energy expenditure and induces thermogenesis in mice (15, 16). To probe the underlying mechanism of weight gain in our study, we assessed energy intake as described previously (31, 32). Mice were housed in metabolic cages to allow quantitative measurements of food and water intake and fecal and urine output. Feces were collected and combusted using a bomb calorimeter to determine fecal energy content and to calculate calories absorbed and digestive efficiency. The results indicate that NR-induced deflection of weight gain was not due to significant differences in food consumption (Fig. 5A), calories absorbed (Fig. 5B), or digestive efficiency (Fig. 5C). However, an NR-dependent reduction in energy efficiency, the rate of weight gain per calorie absorbed throughout the intervention period of time, was observed (Fig. 5D; *P* = 0.05). Since both groups consumed the same amount of food, absorbed the same number of calories, and exhibited similar digestive efficiencies, we conclude that NR-treated mice gain less weight due to an increased energy expenditure. In other words, NR-treated mice burn more calories than control mice, thus gaining less weight. Our results are consistent with dietary NR supplementation reducing HFD-induced weight gain likely through an increase in energy expenditure.

### NR-conditioned FMT increases energy expenditure.

**FIG 6:**
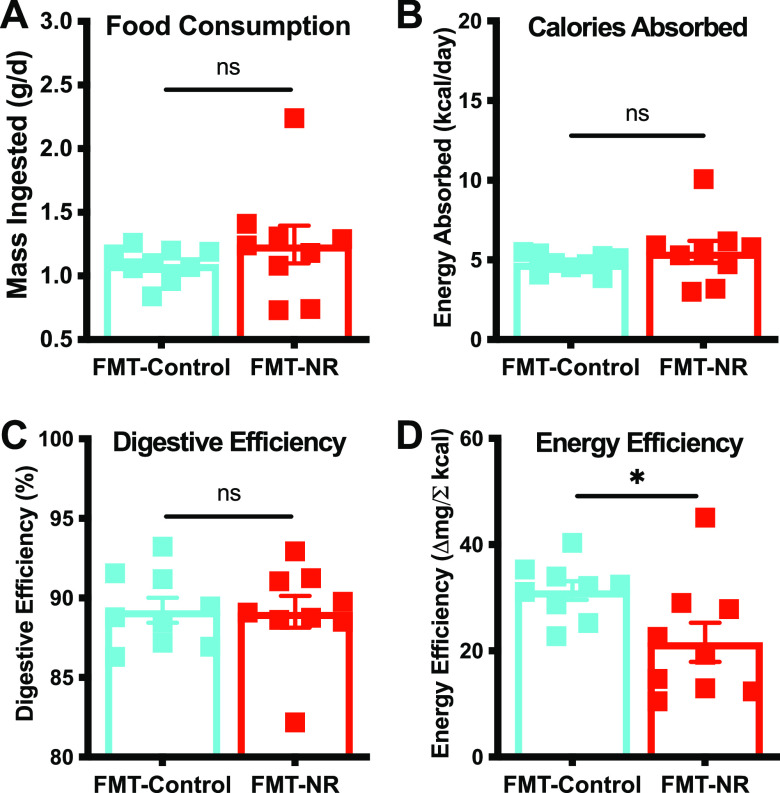
NR-conditioned fecal material transfer decreases energy efficiency. To understand the underlying mechanism of weight gain, mice were housed in metabolic cages and feces were subjected to bomb calorimetry. No significant differences between treatment groups were found in food consumption (A), energy absorbed (B), or digestive efficiency (C). (D) FMT-NR-treated mice (red) exhibited a statistically significant decrease in energy efficiency compared to FMT-control mice (cyan). This suggests that the reduction in weight gain is likely due to an increase in energy expenditure. Data are represented as the mean ± SEM. Data were analyzed by an unpaired *t* test with Welch’s correction (*, *P* < 0.05).

While NR supplementation has been shown to increase energy expenditure in mice (15, 16), the relationship between the gut microbiome and energy expenditure is not well understood. Our laboratory demonstrated previously that an FMT from risperidone-treated mice into naive recipients is sufficient to suppress energy expenditure through a reduction in nonaerobic resting metabolic rate (19). In contrast, the short-chain fatty acid (SCFA) butyrate prevents diet-induced obesity and insulin resistance and is thought to do so by promoting energy expenditure (33). Because NR treatment led to an enrichment of butyrate producers, we hypothesized that the NR-conditioned microbiota contributes to the amelioration of HFD-induced weight gain via increased energy expenditure. Following the same methodology as that described in the legend to Fig. 5, we employed metabolic cages and bomb calorimetry to measure energy efficiency in the FMT cohort. We did not find significant differences between treatment groups in food consumption (Fig. 6A), calories absorbed (Fig. 6B), or digestive efficiency (Fig. 6C). However, FMT-NR-treated mice exhibited a statistically significant decrease in energy efficiency compared to FMT-control mice (Fig. 6D), suggesting that the reduction in HFD-induced weight gain is likely due to an increase in energy expenditure, similar to that with dietary NR supplementation (Fig. 5D). These results suggest that NR-conditioned FMT is sufficient to increase energy expenditure and deflect HFD-induced weight gain.

### NR-conditioned FMT alters NADH/NAD^+^ ratios in the cecum.

**FIG 7:**
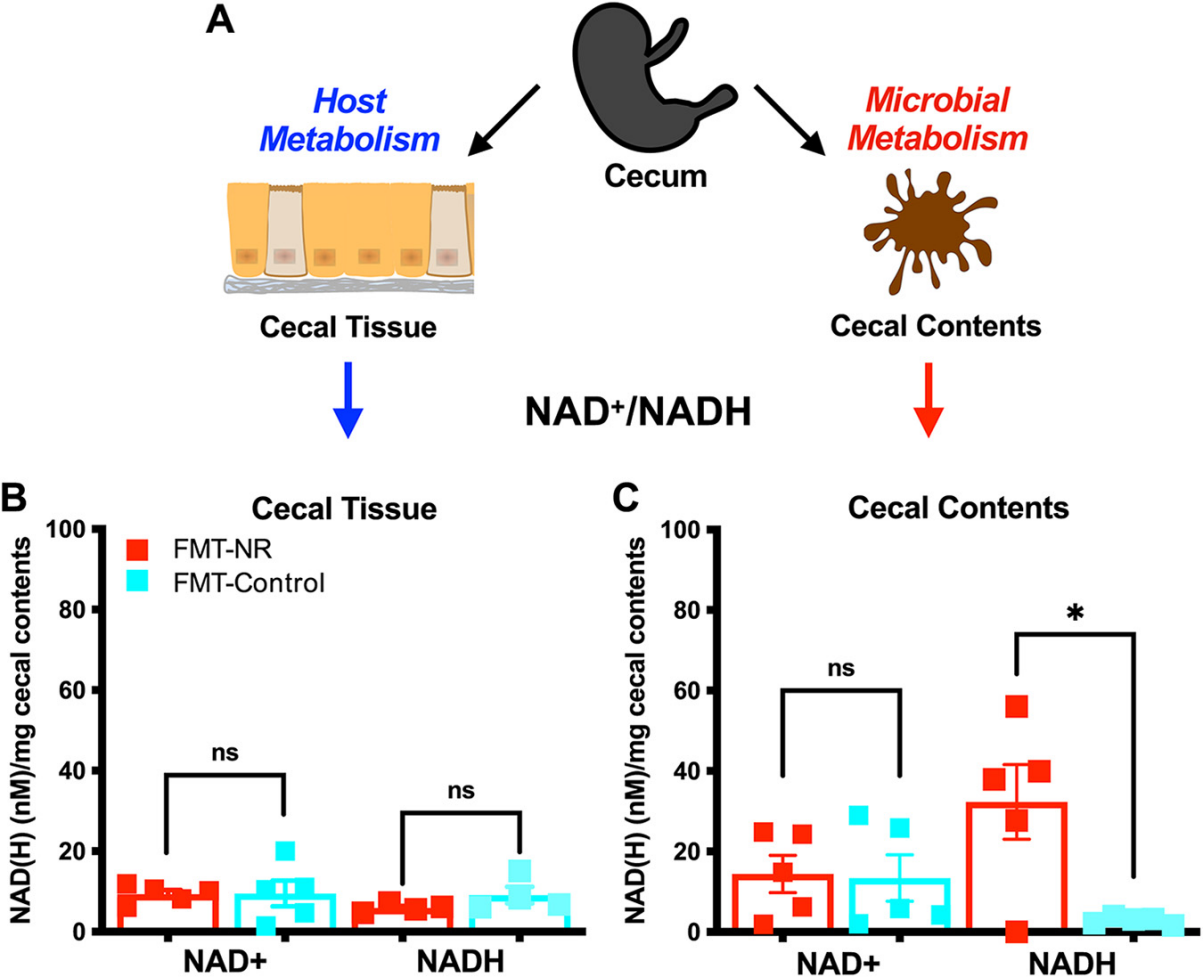
NR-conditioned fecal material transfer alters NAD^+^/NADH ratios in the cecum. (A) Mouse ceca were harvested, flash-frozen in liquid nitrogen, and stored at −80°C until processed. Cecal contents were separated from the tissue, and these were processed separately. Samples were equally split in half. To extract NAD^+^ and NADH metabolites, acidic and basic extractions were performed, respectively, from both the cecal tissue and the cecal contents. (B) FMT-NR-treated mice (red squares) and FMT-control mice (cyan squares) exhibited similar NAD+ levels in the cecal tissue. FMT-NR- treated mice (red squares) and FMT-control mice (cyan squares) exhibited similar NADH levels in the cecal tissue. (C) FMT-NR-treated mice (red squares) and FMT-control mice (cyan squares) exhibited similar NAD+ levels in the cecal contents. FMT-NR-treated mice (red squares) exhibited a significant increase in NADH levels in the cecal contents compared to FMT-control mice (cyan squares). Data are represented as the mean ± SEM. Data were analyzed by an unpaired *t* test with Welch’s correction (*, *P* < 0.05).

The gut microbiota metabolizes dietary components and xenobiotics and produces SCFAs, vitamins, and other metabolites that are taken up by the host. A recent study showed that the colonic microbiota influences systemic NAD^+^ metabolism by boosting the hepatic NAD^+^ biosynthesis in mice treated with NR (34). Furthermore, NR, which can be metabolized and produced by bacteria (13, 35–38), increases NAD^+^ levels in metabolic tissues and subsequently improves host metabolism (15, 25, 26). Thus, to evaluate how FMT-NR influences host and microbial metabolism, we assessed NAD^+^ and NADH levels in the gut. We selected the cecum because it has a high bacterial density and serves as a major site of dietary fermentation, which makes it an important metabolic organ (39–41). We harvested the ceca, separated the cecal contents from the tissue, and assessed the NAD^+^ and NADH levels of the cecal tissue (as a readout for host metabolism) and cecal contents (as a readout for bacterial metabolism) using a colorimetric kit (see Materials and Methods) (Fig. 7A). We found no statistical differences in the NAD^+^ or NADH levels in the cecal tissue (Fig. 7B). In the cecal contents, there were no significant differences in NAD^+^ levels between treatment groups; however, there was a significant increase in the NADH level in FMT-NR mice (Fig. 7C). These results demonstrate that daily NR-conditioned fecal material transfer is capable of altering microbial metabolism in the cecum.

## DISCUSSION

In this study, we employed a diet-induced obesity (DIO) mouse model to investigate whether the benefits of dietary nicotinamide riboside (NR) supplementation are, in part, mediated through the gut microbiota. For decades, mouse DIO models have been used, as they closely mimic the pathophysiological progression of obesity in humans. We used the D12492 diet to induce obesity in mice, which has 60% of the dietary energy coming from fat. Both dietary NR supplementation and NR-conditioned fecal material were sufficient to deflect weight gain induced by the high-fat diet. The observed reductions, in both experiments, are attributable to reduced energy efficiency (Fig. 5D and 6D): there was no significant difference in food intake, calories absorbed, or digestive efficiency. Thus, reduced weight gain in response to NR treatment or to the FMT from NR donors occurs by an increase in energy expenditure for the host. Furthermore, because there were no significant differences observed for SCFAs in the FMT recipients, the observed weight reduction may not depend on the SCFA metabolites *per se* but on other factors attributable to altered microbial composition. Overall, the NR-conditioned microbiota appears to be a reservoir for potential therapeutics to curb diet-induced obesity and should be explored in further detail.

Fecal material transfers (FMTs) have gained popularity due to their capability of restoring a dysbiotic gut microbiota. However, the International Scientific Association for Probiotics and Prebiotics (ISAPP) does not recognize the procedure as a probiotic due to their uncharacterized and potentially hazardous composition (42, 43). In this study, we employed an FMT approach to show that the gut microbiome contributes to the NR-dependent reduction of HFD-induced weight gain. However, deep sequencing of the input and output stool revealed valuable information about the taxonomic and functional capacity of the NR-conditioned microbiota that could lead to the discovery of a defined probiotic to curb HFD-induced weight gain. For example, NR supplementation leads to enrichment of butyrate-producing *Firmicutes* (Fig. 2B; see Fig. S3 in the supplemental material). Bacterially produced butyrate is the primary energy source of colonocytes (44), plays a role in cell differentiation (45, 46), is a histone deacetylase (HDAC) inhibitor (47), and strengthens the epithelial gut barrier (48), among other functions. Further, we found an enrichment of *Romboutsia* (Fig. 2B) in the feces of NR-treated mice, and *Romboutsia* produces butyrate (49) and was found enriched when HFD-induced weight gain was deflected with hydroxysafflor yellow A (safflomin A) (50). Therefore, *Romboutsia* spp. could represent a probiotic candidate to ameliorate weight gain induced by HFD, perhaps through the production of butyrate. Indeed, NR supplementation led to increased SCFAs found in feces (Fig. 2C). However, the FMT-NR mice did not exhibit increased levels of fecal butyrate or other SCFAs but displayed increased NADH levels in the cecal contents (Fig. 7C). Overall, the results suggest that distinct gut microbial communities following NR treatment or FMT from NR-treated donors alter gut flora to affect host energy efficiency and corresponding changes in body mass.

Further mining of our metagenomics data sets allows us to speculate on additional biological features that may play a role in reducing diet-induced weight gain. For instance, the feces of NR-treated mice are enriched for KEGG identifiers K02052 and K02055, which are annotated as a putative spermidine/putrescine ABC transporters. Spermidine and putrescine are polyamines that can be synthesized *de novo* by gut bacteria or host cells or can be acquired through the diet (51, 52). In fact, high levels of polyamines in metabolically active tissues can stimulate energy expenditure and ameliorate diet-induced obesity (52–56). Further, administration of spermidine into diet-induced obesity mouse models effectively decreases weight gain and ameliorates hepatic steatosis (52–54). Therefore, we speculate that NR supplementation reshapes the gut microbiome such that bacteria can produce and metabolize polyamines, which might contribute to the reduced weight gain phenotype. In this context, bacterially derived polyamines and respective transporter genes could be important bacterium-specific targets for the development of antiobesity therapeutics.

Another example of NR-specific biological features that imply bacterially derived molecules as potential contributors to the reduced weight gain phenotype are the genes *nisK*, *nisR*, *nukF*, and *nukG*. The *nis* genes encode a two-component system (TCS) for the autoregulation of nisin production, while the *nuk* genes encode members of the self-protection system against the lantibiotic nukacin ISK-1. Both nisin and nukacin ISK-1 are class I bacteriocins, or lantibiotics, known to have antimicrobial activity by inhibiting cell wall synthesis (57). We found an NR-dependent enrichment of these genes (or its close relatives), which could indicate a survival advantage to sensing and producing lantibiotics in the NR-treated gut. Several studies have suggested the use of lantibiotics as tools for manipulation of dysbiotic microbiota that arises with metabolic diseases, such as obesity and T2D (58–61). Therefore, lantibiotics and lantibiotic-producing bacteria could be used to reshape the gut microbiota into a nondysbiotic state. Together, our metagenomics data support the notion of NR reshaping the microbiota to produce small molecules that further interact with the microbiota and the host to alter metabolism and physiology.

Obesity arises from a chronic imbalance in energy, which in part can be modulated by the gut microbiome through the metabolism of polysaccharides and other substrates, thus contributing to digestive efficiency, a component of energy balance of the host (62). In mice, the cecum, which accounts for approximately 1% of body mass (63), is a major site for bacterial fermentation and production of SCFAs and other metabolites. Furthermore, HFD feeding reduces cecal mass, alters bacterial physiology (64), and reduces NAD^+^ levels, thus affecting energy homeostasis in key metabolic tissues (15). In this study, we used NAD(H) metabolites as a proxy of host and microbial metabolism in the cecal tissue and cecal contents, respectively. While FMT-NR treatment does not affect NAD^+^ or NADH levels of the cecum tissue, in the cecal contents of FMT-NR mice, we found an increase in NADH levels, which suggests an altered microbial metabolism. Our energy flux data do not show any differences in digestive efficiency between FMT-NR and FMT-control mice, but FMT-NR mice do show a reduction in energy efficiency. The fact that NR-conditioned microbiota is sufficient to deflect weight gain and increase energy expenditure demonstrates that the gut microbiota may represent a thermogenic biomass itself (62). However, our experiments do not distinguish between the host or the bacteria burning additional calories following the NR-conditioned FMT. Additional experimentation is required to establish the factors responsible for the change in energy expenditure. Nevertheless, our results support the hypothesis that bacterial energy flux is changing and that modulation of bacterial energy expenditure contributes to the reduction in HFD-induced weight gain. Furthermore, we conclude that the NR-conditioned fecal and cecal microbiota may be a reservoir for potential therapeutics that can be used to enhance the beneficial effects of NR and/or to curb diet-induced obesity.

## MATERIALS AND METHODS

### Animal experiments. (i) Dietary NR supplementation experiment.

Six-week-old male C57BL/6J mice from the Jackson Laboratory were individually housed at the Medical College of Wisconsin (MCW) in a standard 12-h dark–12-h light cycle with *ad libitum* access to a 60% high-fat diet (Research Diets; D12492) and sterile water. Mice were weight matched, randomized, and divided into two treatment groups, control or NR. To prepare NR food, 4 g NR chloride (Niagen; ChromaDex, Inc.) was dissolved in 40 mL of sterile water and mixed with 1 kg of diet. Food was placed in ice cube trays and frozen at −80°C. To control for food processing, 1 kg of HFD control food was mixed with 40 mL of water and processed in the same fashion without the addition of NR.

### (ii) Fecal material transfer experiment.

Six-week-old C57BL/6J male mice from the Jackson Laboratory were individually housed at MCW in a standard 12-h dark–12-h light cycle with *ad libitum* access to a 60% high-fat diet (Research Diets; D12492) and sterile water. Mice were weight matched, randomized, and divided into two treatment groups, FMT-NR and FMT-control.

Fresh fecal slurries were prepared daily using 0.1 g of donor stool/mL sterile PBS, which was homogenized and spun down at 1,000 × g for 5 min. One hundred microliters of supernatant was transferred via oral gavage.

### (iii) Ethical approval.

All procedures were approved by the Medical College of Wisconsin Animal Care and Use Committee in compliance with the National Institutes of Health *Guide for the Care and Use of Laboratory Animals*. The NR-HFD supplementation experiment was performed once due to the time required to induce statistically significant differences in weight relative to controls. These mice served as fecal donors for naive recipients in the FMT experiment.

### Fasting blood glucose.

After the mice were fasted for 6 h, a small incision was made in the tail vein and a drop of blood was applied to a glucometer (OneTouch Ultra 2; LifeScan) to determine blood glucose concentration.

### DNA extraction and metagenomics sequencing and analyses. (i) Fecal collection and DNA extraction.

Mice were individually placed in sterile 500-mL beakers for 2 h to collect fresh stool. Eight pellets of stool were subjected to DNA extraction using the PowerSoil DNA isolation kit (QIAGEN) by following the manufacturer's instructions.

### (ii) Shotgun metagenomics.

Twenty-four fecal samples from the dietary and FMT experiments were subjected to 2 × 150 paired-end shotgun metagenomics sequencing using the Illumina NovaSeq platform by the University of Wisconsin–Madison Biotechnology Center (Table 1). Generated reads were trimmed using Trimmomatic (version 0.38) (65) to remove low-quality reads. Forward and reverse reads were merged using Illumina-utils (version 1.5.0) (66) and then subsampled to the lowest number of reads per sample using Mothur (version 1.35.1) (67). Reads from all samples were coassembled using MegaHit (version 1.1.1) (68, 69), resulting in a single contigs file. The contigs file was simplified using the anvi-script-reformat-fasta, which is part of Anvi’o (version 5.4) (70), and was then indexed using Bowtie2 (version 2.2.8) (71). Individual sample read files were mapped back to the contigs file, also using Bowtie2 (71) and samtools (version 1.3) (72). The resulting BAM files provided sample information in comparison to the whole. Anvi’o was used to create a contigs database, which calculated k-mer frequencies for each contig, identified open reading frames (ORFs) using Prodigal (73), and split the contigs into 20,000-bp regions (70). Anvi’o was used to run hidden Markov models (HMMs), which searched for single-copy genes to predict the number of total genomes present (70). Protein annotation was added using COGs (74), Pfam (75), and InterproScan (76). Taxonomic identity was added using KAIJU (version 1.7.2) (77). Profile databases were generated for each sample using Anvi’o; these databases store sample-specific information about the contigs, such as mean coverage, standard deviation of coverage, and single nucleotide variant information (70). The individual profiles were merged into one profile with hierarchical clustering. Downstream visualization, binning, and analyses were performed using Anvi’o (70). Automatic binning of splits into population genomes was performed using CONCOCT (version 0.4.1) (78). Bins (>2-Mb long) were manually curated to decrease redundancy (<10%) and increase completion (>50%) of metagenome-assembled genomes (MAGs). We assessed the quality of these MAGs using the four previously published bacterial single-copy core gene collections found in Anvi’o (79).

**TABLE 1:** Metagenomics analysis pipeline results^*a*^

Step	Value for:	
	Dietary expt (12 samples from day 168)	FMT expt (12 samples from day 35)
Total raw reads (no.)	171,675,983	184,716,444
Surviving reads (no.)	165,049,327	177,226,972
Merged reads (no.)	70,902,004	75,781,814
Subsampled merged reads (no. of reads/sample)	5,420,903	4,990,676
Total contigs (no.)	78,360	69,790
*N*_50_ (bp)	6,873	6,657
Genomes (no.)	88	78

a Total raw reads were trimmed using Trimmomatic to remove low-quality reads. The surviving forward and reverse reads were merged using Illumina-utils and then subsampled to the lowest number of reads per sample using Mothur. After coassembly, HMMs were implemented to estimate the number of unique genomes present in each data set.

### (iii) LEfSe analysis.

MAGs were subjected to the LDA effect size (LEfSe) (80) algorithm to identify discriminant features. The alpha value for the factorial Kruskal-Wallis test among classes and for the pairwise Wilcoxon test between subclasses was set to 0.05. The threshold on the logarithmic LDA score for discriminative features was set to 2.0. The analysis was run on the https://huttenhower.sph.harvard.edu/galaxy/ site.

### (iv) KEGG functional analysis.

The mean coverage of genes and functional annotations were exported from Anvi’o as tables. Using in-house python scripts, we matched the mean coverage to the corresponding gene annotations (KEGG database) and normalized it to mapped reads per sample. The data were formatted to have the features (pathways and KOs) as rows and the subjects as columns. We removed category 3 pathways that did not belong to bacterial annotations, and the remaining ones were analyzed using LEfSe. The KOs from enriched pathways were analyzed using LEfSe.

### (v) Cytoscape network analysis.

The discriminant KOs identified by LEfSe were used to generate a network analysis using the following python modules: pandas, numpy, matplotlib.pyplot, and network. The network was reformatted and imported into Cytoscape, where the compound spring embedded (CoSE) layout was applied. The node size represents the LDA score (LEfSe analysis), and the edge width represents the *r* value (Pearson’s correlation). We set a cutoff of 0.7 for the *r* values to decrease the number of edges and improve the visualization of the network.

### (vi) Butyrate KO analysis.

We annotated microbial genes using Kyoto Encyclopedia of Genes and Genomes (KEGG) orthology (KEGG orthology genes [KOs]). To identify discriminant KOs from butyrate production pathways in the enriched MAGs, we implemented LEfSe on the following KOs: K00004, K00019, K00074, K00100, K00132, K00169, K00170, K00171, K00172, K00174, K00175, K00209, K00239, K00240, K00241, K00244, K00246, K00626, K00634, K00656, K00823, K00929, K01027, K01029, K01034, K01035, K01039, K01040, K01580, K01615, K01640, K01641, K01715, K03366, K03737, K04072, K04073, K07246, K07250, K07516, K14534, K17865, K18118, K18119, K18120, K18121, K18122, K18366, K19709, K20509, K23351, K23352, K00248, K03522, and K03521.

### NAD(H) levels of cecal tissue and cecal contents.

Ceca were harvested, flash-frozen in liquid nitrogen, and stored at −80°C until processed. Cecal tissue was separated from the cecal contents by pushing out the feces and washing the tissue in sterile ice-cold phosphate-buffered saline (PBS). Cecal tissue and cecal content samples were split in half, and these metabolites were extracted in either acidic (NAD^+^) or basic (NADH) buffers by following the manufacturer’s protocol (EnzyChrom NAD/NADH; BioAssay Systems).

### Energy flux assessment.

Mice were singly housed in metabolic cages (Nalgene) for 3 to 4 days to monitor daily water, food intake, and urine and fecal output. Feces were collected and combusted in a bomb calorimeter to calculate the digestive efficiency and energy efficiency.

The caloric densities of food and feces were determined using a 50 mg semi-micro bomb calorimeter (19, 31). The following formula was used to calculate energy absorption:

calories absorbed = energy consumed − fecal energy

Energy consumed was calculated by multiplying the dry mass of food by the caloric density of dry food, whereas fecal energy was calculated by multiplying the dry mass of feces by the caloric density of desiccated feces.

The following formulas were used to calculate digestive and energy efficiencies:

$$\text{digestive efficiency }=\;\frac{\text{calories absorbed}}{\text{calories consumed}}$$

$$\text{energy efficiency }=\frac{\Delta \text{body mass}}{\sum \text{calories absorbed}}$$

### Measurement of SCFAs. (i) Sample preparation.

Samples were analyzed for short-chain fatty acids (SCFAs) using a modified version of a previously described protocol (81). Approximately 50 mg of fecal material was weighed into a tared 1.5-mL Eppendorf tube. Two hundred forty microliters of water and 560 μL of acetonitrile containing internal standards (500 μM D4-acetic acid, 250 μM D7-butyric acid, and 6.25 μM D11-hexanoic acid) were added to each sample. Samples were homogenized by probe sonication for 20 s using a Branson 450 Sonifier set at power level 4, duty cycle 40%. Samples were centrifuged at 15,000 relative centrifugal force (rcf) at 4°C for 10 min, and 80 μL of supernatant was transferred to a 1.8-mL glass autosampler vial. To this vial was added 15 μL of 200 mM 3-nitrophenylhydrazine (3-NPH) in 1:1 acetonitrile-water and 15 μL of 120 mM 1-ethyl-3-(3-dimethylaminopropyl)carbodiimide in 1:1 acetonitrile-water with 6% pyridine. Samples were capped, vortexed, and placed in a warming oven at 40°C for 30 min. Once derivatization was complete, the samples were cooled and diluted by the addition of 800 μL of 90/10 water-acetonitrile; the samples were recapped and submitted to LC-MS analysis. A standard curve was prepared identically to that for cecal samples, with substitution of 80 μL volatile fatty acid mix (Sigma CRM46975) diluted to concentrations ranging from 3 μM to 3,000 μM.

### (ii) LC-MS analysis.

Samples were analyzed using an Agilent (Santa Clara, CA) 1290 liquid chromatograph (LC) coupled to an Agilent 6490 triple quadrupole mass spectrometer (MS). The chromatographic column was a Waters (Milford, MA) HSS T3, 2.1 mm by 100 mm, with a 1.7-μm particle size. Mobile phase A was 0.1% formic acid in water; mobile phase B was 0.1% formic acid in methanol. The gradient was as follows: linear ramp from 15% to 80% B from 0 to 12 min; step to 100% B from 12 to 12.1 min; hold 100% B from 12.1 to 16 min; step to 15% B from 16 to 16.1 min; hold 15% B from 16.1 to 20 min. The injection volume was 5 μL, and the column temperature was 55°C. MS parameters were as follows: gas temp, 325°C; gas flow, 10 L/min; nebulizer, 40 lb/in^2^; capillary voltage, 4,000 V; scan type, MRM; negative-ion mode, delta EMV 600. Quantitation was performed using Agilent MassHunter Quantitative Analysis software version 8.0 by measuring the ratio of peak area of the 3-NPH derivatized SCFA species to its closest internal standard (by retention time). Linear standard curves were used to estimate SCFA concentrations in the extract, which were normalized to the measured mass of fecal contents.

### Statistics.

Weight gain progression was analyzed using two-way analysis of variance (ANOVA) with repeated measures, followed by Tukey’s multiple-comparison procedures. We implemented an unpaired *t* test with Welch’s correction for weight gain, body mass comparisons, metabolic assessment, and NAD(H) levels. Significance was assigned at a *P* of <0.05 (indicated by an asterisk in figures). Data are reported as the mean ± standard error of the mean (SEM).

### Data availability.

Raw reads for shotgun metagenomes are available in the Sequence Read Archive under accession no. PRJNA704567. The bioinformatics workflow document and scripts used can be found in the GitHub repository at https://github.com/val92loz/HFD-NR_microbiome.git.

10.1128/mSystems.00230-21.7 TABLE S3 Results of LEfSe analysis done on all KOs from the dietary supplementation experiment. Download Table S3, PDF file, 0.1 MB. Copyright © 2022 Lozada-Fernández et al. 2022 Lozada-Fernández et al. https://creativecommons.org/licenses/by/4.0/ This content is distributed under the terms of the Creative Commons Attribution 4.0 International license.

10.1128/mSystems.00230-21.8 TABLE S4 Results of LEfSe analysis done on KOs found within the enriched pathways from the dietary supplementation experiment. Download Table S4, PDF file, 0.1 MB. Copyright © 2022 Lozada-Fernández et al. 2022 Lozada-Fernández et al. https://creativecommons.org/licenses/by/4.0/ This content is distributed under the terms of the Creative Commons Attribution 4.0 International license.

10.1128/mSystems.00230-21.9 TABLE S5 Results of LEfSe analysis done on all KOs from the FMT experiment. Download Table S5, PDF file, 0.1 MB. Copyright © 2022 Lozada-Fernández et al. 2022 Lozada-Fernández et al. https://creativecommons.org/licenses/by/4.0/ This content is distributed under the terms of the Creative Commons Attribution 4.0 International license.

10.1128/mSystems.00230-21.10 TABLE S6 Results of LEfSe analysis done on KOs found within the enriched pathways from the FMT experiment. Download Table S6, PDF file, 0.1 MB. Copyright © 2022 Lozada-Fernández et al. 2022 Lozada-Fernández et al. https://creativecommons.org/licenses/by/4.0/ This content is distributed under the terms of the Creative Commons Attribution 4.0 International license.
