# Supplementary material for: Nicotinamide Riboside-Conditioned Microbiota Deflects High-Fat Diet-Induced Weight Gain in Mice
Source: mSystems. 2022 Jan 25;7(1):e00230-21. doi: 10.1128/msystems.00230-21 (PMC8788325; doi:10.1128/msystems.00230-21)
Supplement: FIG S1 [file msystems.00230-21-sf001.pdf]

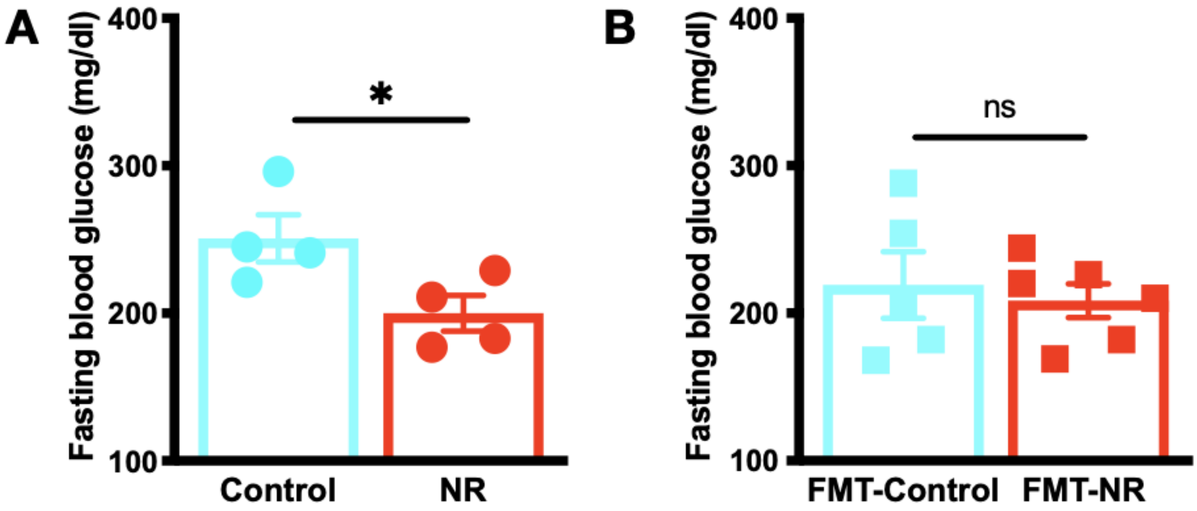

Supplementary Figure 1. NR treatment, but not FMT-NR, reduces fasting blood glucose levels. Mice were fasted for hours. Blood was drawn from tail vein and glucose was measured using a glucometer. A) NR-treated mice exhibited lower fasting blood glucose levels compared to control mice. B) In contrast, no significant differences in fasting blood glucose levels were found between FMT-NR and FMT-Control. Data represented as mean ± sem. *Analyzed by Two-tailed Student's t-test. p* < 0.05.*
