# Supplementary material for: Nicotinamide Riboside-Conditioned Microbiota Deflects High-Fat Diet-Induced Weight Gain in Mice
Source: mSystems. 2022 Jan 25;7(1):e00230-21. doi: 10.1128/msystems.00230-21 (PMC8788325; doi:10.1128/msystems.00230-21)
Supplement: TABLE S1 [file msystems.00230-21-st001.pdf]

bins	total_length	num_contigs	GC_content	%completion	%redundancy
Actinobacteria_phylum	4347112	1308	57.8638918	48.92086331	9.352517986
Akkermansia_mucinophila	2731069	75	54.966319	98.56115108	1.438848921
Alistipes	1988539	26	59.0561606	97.84172662	0
Anaerotruncus_G3	1155357	260	57.2750212	51.07913669	7.913669065
Anaerotruncus_G3_2	3637814	757	54.5932673	92.08633094	5.755395683
Atopobium	1984597	28	58.7488875	97.84172662	1.438848921
Bacteroides	7608478	394	45.6366848	63.30935252	7.194244604
Bacteroides_2	3255325	539	49.0410302	56.11510791	7.913669065
Butyrivibrio	4110392	1324	49.1979418	51.07913669	7.913669065
Clostridiales	2867643	644	54.4162515	53.95683453	6.474820144
Clostridiales_2	556848	195	50.6067838	51.07913669	7.194244604
Clostridiales_3	3162692	507	52.3178016	48.20143885	7.913669065
Clostridiales_4	2769105	694	52.8389457	59.71223022	6.474820144
Clostridium	2884440	741	51.3708208	51.07913669	8.633093525
Clostridium_2	2675450	526	54.7760745	56.83453237	9.352517986
Clostridium_3	3228928	1039	47.8460528	48.20143885	8.633093525
Clostridium_4	2568983	1119	47.4732064	50.35971223	8.633093525
Clostridium_5	3610375	1871	48.972743	54.67625899	9.352517986
Clostridium_6	3631221	966	52.5372662	44.60431655	9.352517986
Clostridium_ASF356	1408669	709	31.1247089	56.83453237	2.158273381
Clostridium_ASF356_2	1096156	211	50.1107817	79.13669065	0.71942446
Clostridium_ASF502	3817244	254	49.2687243	92.08633094	5.755395683
Dorea	2975792	1453	46.323789	53.95683453	9.352517986
Dorea_2	4714965	706	47.1285823	73.38129496	5.755395683
Dorea_3	4022761	1561	47.870451	50.35971223	9.352517986
Enterorhabdus_caecimuris	2102284	388	65.6612276	51.79856115	7.194244604
Enterorhabdus_caecimuris_2	2353074	708	65.4412992	64.74820144	6.474820144
Enterorhabdus_mucosicola	2260354	384	65.8059121	50.35971223	7.913669065
Erysipelatoclostridium	2855887	277	28.5198179	97.84172662	5.035971223
Eubacterium	1718053	248	46.3116985	53.23741007	8.633093525
Eubacterium_2	1890174	816	46.9848423	50.35971223	5.755395683
Eubacterium_3	4106395	774	49.0487879	58.27338129	6.474820144
Eubacterium_4	3520257	1233	46.5285663	38.1294964	9.352517986
Firmicutes	5562598	1504	51.2313824	55.39568345	8.633093525
Firmicutes_2	4453192	1932	46.2590398	51.07913669	9.352517986
Firmicutes_3	3776952	1602	52.3435197	50.35971223	9.352517986
Firmicutes_ASF500	2026697	615	60.4653977	66.18705036	7.194244604
Firmicutes_ASF500_2	874035	219	61.8685871	52.51798561	7.194244604
Firmicutes_ASF500_3	1761692	403	60.5298429	51.07913669	2.158273381
Firmicutes_ASF500_4	4303837	1520	58.2060671	49.64028777	9.352517986
Firmicutes_ASF500_5	2092494	666	58.3372821	38.84892086	9.352517986
Firmicutes_M10-2	2144684	128	41.9948909	96.4028777	5.035971223
Flavonifractor_plautii	2062591	362	59.6646504	71.94244604	8.633093525
Flavonifractor_plautii_2	2270377	566	57.3160354	44.60431655	8.633093525
Hungatella	3609645	1172	51.9536401	51.79856115	9.352517986
Kineothrix_alysoides	2291727	645	49.654547	48.20143885	7.913669065
Lachnoclostridium	2802191	318	51.4829715	84.17266187	7.913669065
Lachnospiraceae	3578970	526	51.3470997	53.95683453	8.633093525
Lachnospiraceae_2	4290866	1562	48.9954909	46.04316547	9.352517986
Lachnospiraceae_bacterium_10-1	2459568	979	47.7305175	49.64028777	9.352517986
Lachnospiraceae_bacterium_10-1_2	2038550	372	51.6053299	48.20143885	8.633093525
Lachnospiraceae_bacterium_28-4	3517581	286	44.3307612	99.28057554	3.597122302
Lachnospiraceae_bacterium_28-4_2	3013253	231	43.4267585	99.28057554	4.316546763
Lachnospiraceae_bacterium_28-4_3	2935151	896	47.3880621	49.64028777	9.352517986
Lachnospiraceae_bacterium_28-4_4	2699588	713	48.0385883	43.16546763	9.352517986
Lachnospiraceae_bacterium_3-1	1946502	268	45.0618547	52.51798561	9.352517986
Lachnospiraceae_bacterium_3-1_2	3116508	832	45.727634	50.35971223	9.352517986
Lachnospiraceae_bacterium_A2	5236671	1938	47.0476826	49.64028777	9.352517986
Lachnospiraceae_bacterium_A4	4836927	533	48.1350215	83.45323741	7.194244604

Lachnospiraceae_bacterium_sp	7194141	2078	48.6488875	53.23741007	9.352517986
Lachnospiraceae_bacterium_sp_2	3986793	1131	50.3636303	49.64028777	9.352517986
Lactobacillus	1925116	70	35.0252313	97.84172662	5.035971223
Lactobacillus_reuteri	1565897	698	38.8512647	66.18705036	2.158273381
Lactococcus_lactis	2437172	157	35.0026848	100	2.877697842
Oscillibacter	1064396	152	59.8878307	53.95683453	5.755395683
Oscillibacter_2	1401986	434	57.2242645	56.11510791	9.352517986
Oscillibacter_3	2976102	976	57.1461765	43.16546763	6.474820144
Oscillibacter_4	1478068	178	60.8734965	51.07913669	5.755395683
Oscillibacter_KLE1728	2521099	556	59.3011677	51.79856115	7.913669065
Oscillibacter_bacterium_1-3	2684311	314	60.6832559	88.48920863	7.194244604
Oscillibacter_bacterium_1-3_2	2716630	467	56.5409486	71.94244604	5.755395683
Oscillibacter_bacterium_1-3_3	2707177	645	56.7406657	74.10071942	7.913669065
Oscillibacter_bacterium_1-3_4	2873828	546	57.8295614	50.35971223	9.352517986
Pseudoflavonifractor_capillosus	2473285	560	56.6552801	42.44604317	9.352517986
Romboutsia	2547253	160	27.0882637	99.28057554	4.316546763
Roseburia	2709870	831	49.5353435	53.95683453	9.352517986
Roseburia_2	1618877	131	44.6328918	51.07913669	8.633093525
Ruminiclostridium	1393282	229	52.8399172	59.71223022	7.913669065
Ruminiclostridium_2	2112812	700	54.6707539	52.51798561	8.633093525
Ruminococcus	2629672	606	53.740092	54.67625899	8.633093525
Ruminococcus_2	2402255	994	49.7214183	44.60431655	9.352517986
Unknown_Burkholderiales	1510035	87	48.2159604	84.17266187	0
Unknown_Clostridiales	2991993	332	53.2988668	95.68345324	6.474820144
Unknown_Clostridiales_2	2075026	430	53.2312642	58.27338129	8.633093525
Unknown_Clostridiales_3	3008534	818	48.8221609	57.55395683	9.352517986
Unknown_Lachnospiraceae	3806482	1450	49.4920186	53.95683453	9.352517986
Unknown_Lachnospiraceae_2	3157023	1271	46.4702089	49.64028777	9.352517986
Unknown_Lachnospiraceae_3	2689693	429	45.6397645	51.79856115	9.352517986

Supplementary Table 1. Summary table of MAGs generated from the dietary NR supplementation experiment. Bins were generated by CONCOCT and then manually refined to satisfy a completion of at least 50% or 2 Mbp and a redundancy of no more than 10%.
