# Supplementary figures and images for: Nicotinamide Riboside-Conditioned Microbiota Deflects High-Fat Diet-Induced Weight Gain in Mice
Source: mSystems. 2022 Jan 25;7(1):e00230-21. doi: 10.1128/msystems.00230-21 (PMC8788325; doi:10.1128/msystems.00230-21)

Figure S2

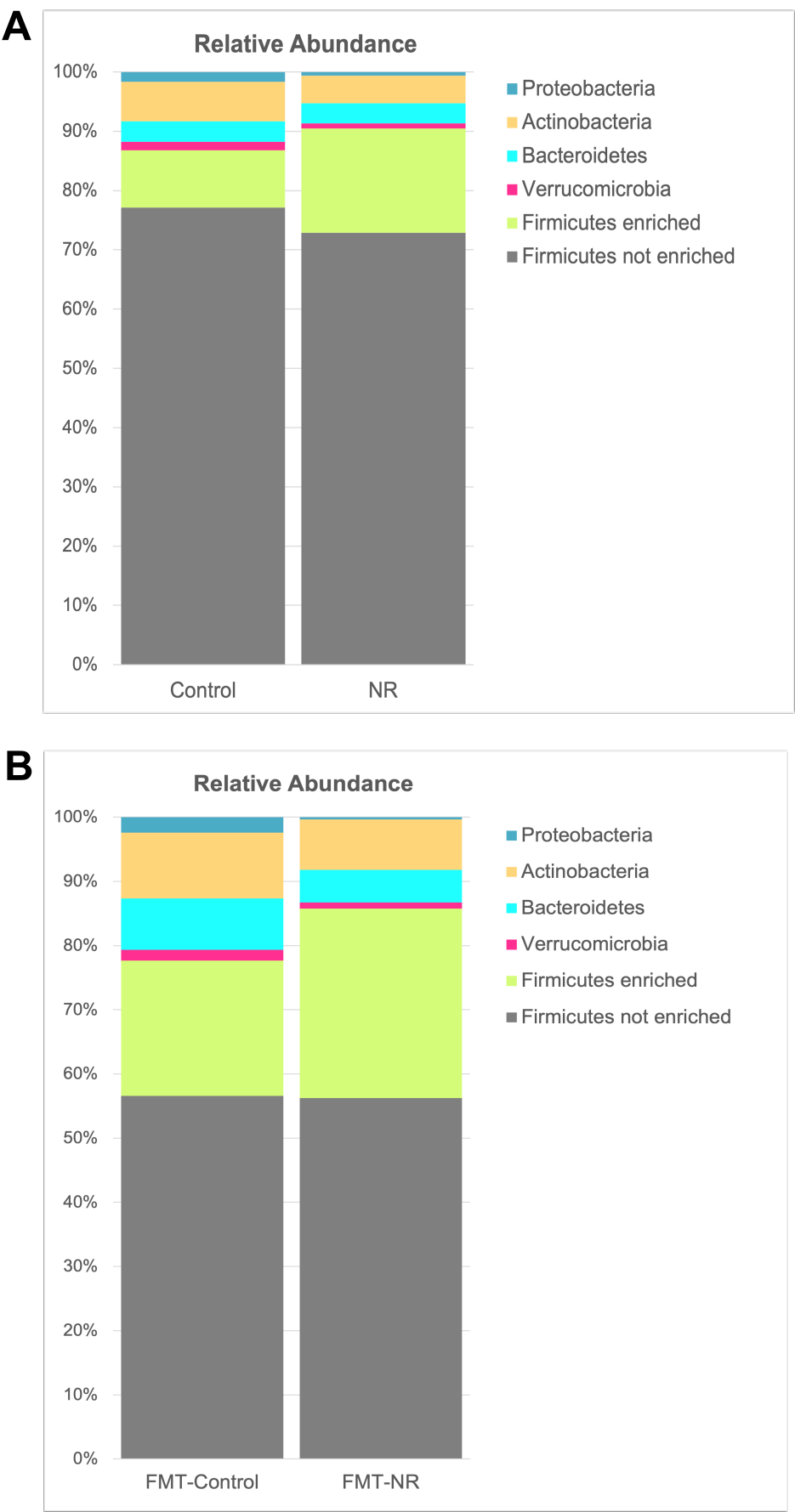

Supplement: FIG S2 [file msystems.00230-21-sf002.pdf]
