# Supplementary material for: Nicotinamide Riboside-Conditioned Microbiota Deflects High-Fat Diet-Induced Weight Gain in Mice
Source: mSystems. 2022 Jan 25;7(1):e00230-21. doi: 10.1128/msystems.00230-21 (PMC8788325; doi:10.1128/msystems.00230-21)
Supplement: FIG S3 [file msystems.00230-21-sf003.pdf]

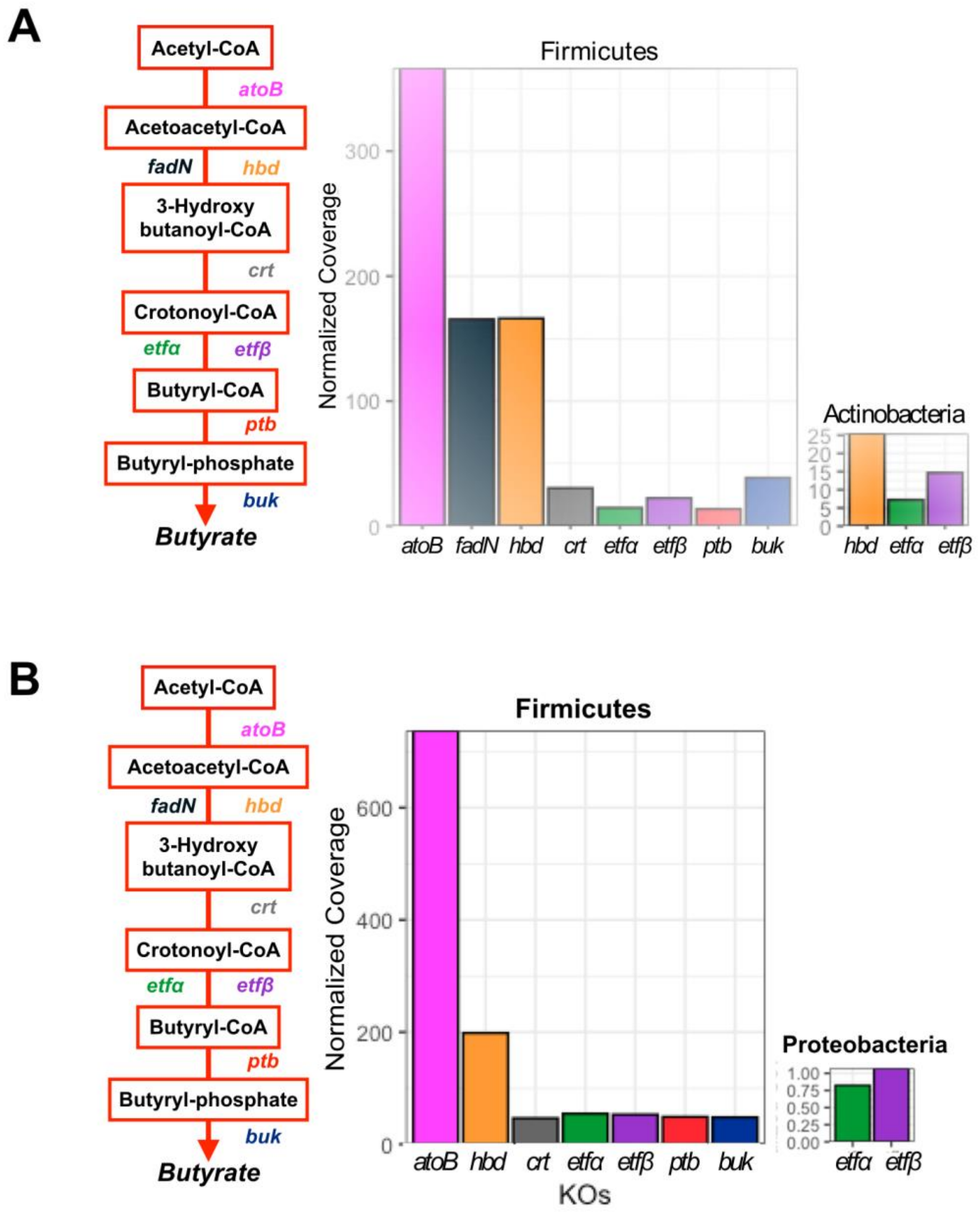

Figure S3. Normalized coverage of genes in the Acetyl-Coa pathway for butyrate synthesis within the 12 enriched MAGs.

A) Dietary experiment. The acetyl-CoA pathway for butyrate synthesis was the most common pathway found within the 12 enriched Firmicutes MAGs in the NR-treated samples. Only one gene was found within the enriched Actinobacteria member, whereas no KOs were found in the enriched Proteobacteria.

B) FMT experiment. The acetyl-CoA pathway for butyrate synthesis was also the most common pathway found within the 19 enriched Firmicutes MAGs in the FMT-NR-treated mice. Only two genes were found within the enriched Proteobacteria member.
