# Supplementary material for: Nicotinamide Riboside-Conditioned Microbiota Deflects High-Fat Diet-Induced Weight Gain in Mice
Source: mSystems. 2022 Jan 25;7(1):e00230-21. doi: 10.1128/msystems.00230-21 (PMC8788325; doi:10.1128/msystems.00230-21)
Supplement: FIG S4 [file msystems.00230-21-sf004.pdf]

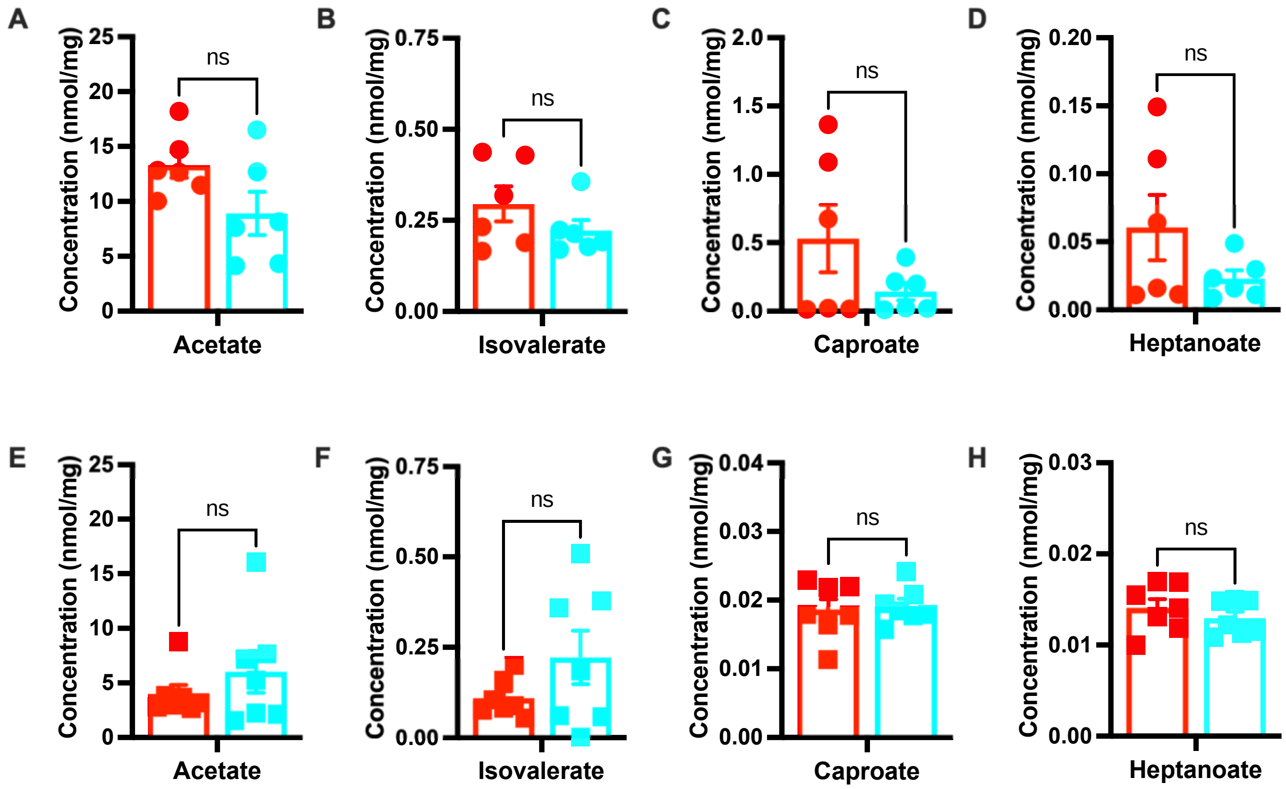

Figure S4. Measurement of SCFA concentration.
Fecal A) acetate, B) isovalerate, C) caproate and D) heptanoate concentrations in the dietary mouse cohort.
Fecal E) acetate, F) isovalerate, G) caproate and H) heptanoate concentrations in the FMT mouse cohort. All values were normalized by mg of feces.
