# Supplementary material for: Nicotinamide Riboside-Conditioned Microbiota Deflects High-Fat Diet-Induced Weight Gain in Mice
Source: mSystems. 2022 Jan 25;7(1):e00230-21. doi: 10.1128/msystems.00230-21 (PMC8788325; doi:10.1128/msystems.00230-21)
Supplement: TABLE S2 [file msystems.00230-21-st002.pdf]

bins	total_length	num_contigs	GC_content	%completion	%redundancy
Actinobacteria	1865004	291	58.0631528	58.2733813	7.91366906
Actinobacteria_2	2205107	502	61.9129056	43.8848921	6.47482014
Akkermansia_mucinophila	2667165	66	54.5447219	98.5611511	1.43884892
Alistipes	1978385	19	59.3117791	98.5611511	0
Anaerotruncus_G3	1844072	263	56.5919124	70.5035971	1.43884892
Anaerotruncus_G3_2	1528234	368	55.6265125	51.0791367	3.5971223
Bacteroides	929434	81	50.6437152	56.1151079	2.87769784
Bacteroides_2	2142308	347	45.8345338	43.1654676	2.15827338
Bacteroides_thetaiotaomicron	5482523	84	42.8932845	67.6258993	1.43884892
Bacteroidetes	3307014	502	48.3327074	44.6043165	7.1942446
Burkholderiales	2093516	440	47.9273725	92.8057554	4.31654676
Clostridium	1130960	288	56.7030386	56.1151079	6.47482014
Clostridium_2	3462115	903	54.0177241	56.1151079	9.35251799
Clostridium_3	3382817	1416	44.8820561	53.2374101	9.35251799
Clostridium_4	1999665	500	56.1571846	50.3597122	9.35251799
Clostridium_ASF356	1161573	159	43.0306897	64.7482014	4.31654676
Clostridium_ASF502	3175651	968	46.899606	36.6906475	2.87769784
Clostridium_symbiosum	2826482	287	52.6644177	68.3453237	6.47482014
Dorea	3381527	283	46.3356744	83.4532374	8.63309353
Dorea_2	2355518	545	46.7500968	56.8345324	8.63309353
Dorea_3	2471501	493	48.0973183	56.1151079	7.91366906
Dorea_4	2487574	978	46.6982809	50.3597122	9.35251799
Enterorhabdus	2304966	416	62.3706015	46.7625899	9.35251799
Enterorhabdus_2	2614150	953	49.3867215	57.5539568	8.63309353
Enterorhabdus_3	1703180	358	62.9717551	49.6402878	7.91366906
Enterorhabdus_caecimuris	1723861	311	64.552901	55.3956835	3.5971223
Enterorhabdus_mucosicola	1851241	422	65.4465418	51.7985612	7.1942446
Eubacterium_14_2	2201771	455	46.7787134	49.6402878	6.47482014
Eubacterium_sp	3830200	1126	46.8813611	48.9208633	9.35251799
Eubacterium_sp_2	1921705	675	48.7355814	57.5539568	7.91366906
Firmicutes	2618620	1269	45.7717195	38.1294964	8.63309353
Firmicutes_2	2901094	1104	55.0375062	51.7985612	9.35251799
Firmicutes_3	3377093	602	40.6576803	79.8561151	6.47482014
Firmicutes_bacterium_ASF500	2026037	360	59.8843374	46.7625899	7.1942446
Firmicutes_bacterium_ASF500_2	2457057	745	61.2296171	49.6402878	7.91366906
Firmicutes_bacterium_ASF500_3	2195691	357	60.7078298	52.5179856	9.35251799
Firmicutes_phylum	2477883	968	50.1346023	56.1151079	9.35251799
Flavonifractor_plautii	2054308	509	59.1964733	46.7625899	8.63309353
Hungatella	2324970	723	51.5127624	39.5683453	9.35251799
Lachnoclostridium	3870392	1347	51.7853065	54.676259	9.35251799
Lachnoclostridium_2	3201366	419	51.8043803	69.0647482	8.63309353
Lachnospiraceae	3883535	714	53.2925433	56.1151079	8.63309353
Lachnospiraceae_2	2796242	578	46.2115069	44.6043165	9.35251799
Lachnospiraceae_3	2960575	1109	50.8301461	53.2374101	9.35251799
Lachnospiraceae_4	3716199	1950	47.3672703	50.3597122	9.35251799
Lachnospiraceae_bacterium	2931337	1066	46.8599388	42.4460432	9.35251799
Lachnospiraceae_bacterium_10-1	2849282	891	47.3275515	45.323741	6.47482014
Lachnospiraceae_bacterium_2	4818023	894	50.957945	46.7625899	6.47482014
Lachnospiraceae_bacterium_28-4_3	2718845	681	47.4432624	43.8848921	9.35251799
Lachnospiraceae_bacterium_28_4	3694220	304	45.5978628	100	5.75539568
Lachnospiraceae_bacterium_28_4_2	3286446	332	44.8291736	89.2086331	6.47482014
Lachnospiraceae_bacterium_3	2644933	727	49.1417933	52.5179856	9.35251799
Lachnospiraceae_bacterium_3-1	2716333	760	44.9732958	65.4676259	7.91366906
Lachnospiraceae_bacterium_3-2	2327820	695	46.8422278	44.6043165	8.63309353
Lachnospiraceae_bacterium_4	3057226	1348	47.1761072	40.2877698	8.63309353
Lachnospiraceae_bacterium_A4	4043008	1545	49.4043165	51.0791367	8.63309353
Lachnospiraceae_bacterium_M18-1	4145251	1104	48.5577671	52.5179856	9.35251799
Lactobacillus_johnsonii	1851971	127	34.462754	97.8417266	7.1942446
Lactococcus_lactis	3201817	467	34.949301	100	6.47482014

Oscillibacter	2515034	375	57.403395	55.3956835	5.75539568
Oscillibacter_1-3	1929835	248	60.2943734	53.2374101	0
Oscillibacter_1-3_2	3595143	983	56.9208575	45.323741	8.63309353
Oscillibacter_1-3_3	1084555	166	62.1538236	58.2733813	2.87769784
Oscillibacter_1-3_4	2494194	630	57.6084786	48.2014388	8.63309353
Oscillibacter_KLE1728	2272510	601	59.3754728	43.1654676	8.63309353
Oscillibacter_sp	1852318	718	58.5675001	50.3597122	9.35251799
Oscillibacter_sp_2	1031404	137	59.8594328	56.1151079	8.63309353
Oscillibacter_sp_3	1029295	270	61.8018354	52.5179856	8.63309353
Pseudoflavonifractor_capillosus	2220618	603	57.4090149	43.1654676	8.63309353
Romboutsia	1008411	165	27.2975842	49.6402878	0
Roseburia	2896260	877	49.7585825	50.3597122	6.47482014
Ruminiclostridium	2577403	688	51.6062966	45.323741	7.91366906
Ruminococcaceae	2076016	730	55.3536029	42.4460432	7.91366906
Ruminococcaceae_2	1905663	506	54.0813301	62.5899281	7.91366906
Ruminococcus	3467712	1187	51.0446227	41.0071942	9.35251799
Subdoligranulum_4_3_54A2FAA	2573457	782	58.8977364	86.3309353	4.31654676
Unknown_Lachnospiraceae	3517042	550	47.292361	46.7625899	9.35251799
Unknown_Ruminococcaceae	1677749	489	57.0951491	56.8345324	7.91366906

Supplementary Table 2. Summary table of MAGs generated from the FMT experiment. Bins were generated by CONCOCT and then manually refined to satisfy a completion of at least 50% or 2 Mbp and a redundancy of no more than 10%.
