# Supplementary material for: Nicotinamide Riboside-Conditioned Microbiota Deflects High-Fat Diet-Induced Weight Gain in Mice
Source: mSystems. 2022 Jan 25;7(1):e00230-21. doi: 10.1128/msystems.00230-21 (PMC8788325; doi:10.1128/msystems.00230-21)
Supplement: TABLE S3 [file msystems.00230-21-st003.pdf]

37 KOs Enriched in NR-treated samples

KOs	Description
K10112	msmX, msmK, malK, sugC, ggtA, msiK; multiple sugar transport system ATP-binding protein
K10117	msmE; raffinose/stachyose/melibiose transport system substrate-binding protein
K10118	msmF; raffinose/stachyose/melibiose transport system permease protein
K10119	msmG; raffinose/stachyose/melibiose transport system permease protein
K03292	TC.GPH; glycoside/pentoside/hexuronide:cation symporter, GPH family
K10439	rbsB; ribose transport system substrate-binding protein
K00172	porG; pyruvate ferredoxin oxidoreductase gamma subunit [EC:1.2.7.1]
K00171	porD; pyruvate ferredoxin oxidoreductase delta subunit [EC:1.2.7.1]
K00170	porB; pyruvate ferredoxin oxidoreductase beta subunit [EC:1.2.7.1]
K05858	PLCB; phosphatidylinositol phospholipase C, beta [EC:3.1.4.11]
K01835	pgm; phosphoglucomutase [EC:5.4.2.2]
K05349	bglX; beta-glucosidase [EC:3.2.1.21]
K00688	PYG, glgP; glycogen phosphorylase [EC:2.4.1.1]
K03407	cheA; two-component system, chemotaxis family, sensor kinase CheA [EC:2.7.13.3]
K02406	fliC; flagellin
K02529	lacI, galR; LacI family transcriptional regulator
K03784	deoD; purine-nucleoside phosphorylase [EC:2.4.2.1]
K07133	K07133; uncharacterized protein
K02405	fliA; RNA polymerase sigma factor for flagellar operon FliA
K02335	polA; DNA polymerase I [EC:2.7.7.7]
K07718	yesM; two-component system, sensor histidine kinase YesM [EC:2.7.13.3]
K18866	vanXY; zinc D-Ala-D-Ala dipeptidase/carboxypeptidase [EC:3.4.13.22 3.4.17.14]
K07260	vanY; zinc D-Ala-D-Ala carboxypeptidase [EC:3.4.17.14]
K07484	K07484; transposase
K07706	agrC, blpH, fsrC; two-component system, LytTR family, sensor histidine kinase AgrC
K02392	flgG; flagellar basal-body rod protein FlgG
K20488	nisR, spaR; two-component system, OmpR family, lantibiotic biosynthesis response regulator NisR/SpaR
K20487	nisK, spaK; two-component system, OmpR family, lantibiotic biosynthesis sensor histidine kinase NisK/SpaK [EC:2.7.13.3]
K02601	nusG; transcription termination/antitermination protein NusG
K07720	yesN; two-component system, response regulator YesN
K02556	motA; chemotaxis protein MotA
K11529	gck, gckA, GLYCTK; glycerate 2-kinase [EC:2.7.1.165]
K00975	glgC; glucose-1-phosphate adenylyltransferase [EC:2.7.7.27]
K02004	ABC.CD.P; putative ABC transport system permease protein
K02003	ABC.CD.A; putative ABC transport system ATP-binding protein
K00169	porA; pyruvate ferredoxin oxidoreductase alpha subunit [EC:1.2.7.1]

9 KOs Enriched in Control-treated samples

KOs	Description
K02113	ATPF1D, atpH; F-type H+-transporting ATPase subunit delta
K01834	PGAM, gpmA; 2,3-bisphosphoglycerate-dependent phosphoglycerate mutase [EC:5.4.2.11]
K01921	ddl; D-alanine-D-alanine ligase [EC:6.3.2.4]
K15580	oppA, mppA; oligopeptide transport system substrate-binding protein
K01689	ENO, eno; enolase [EC:4.2.1.11]
K02794	PTS-Man-EIIB, manX; PTS system, mannose-specific IIB component [EC:2.7.1.191]
K10823	oppF; oligopeptide transport system ATP-binding protein
K01874	MARS, metG; methionyl-tRNA synthetase [EC:6.1.1.10]
K01710	E4.2.1.46, rfbB, rffG; dTDP-glucose 4,6-dehydratase [EC:4.2.1.46]

Supplementary Table 3. Results of LEfSe analysis done on all KOs from the Dietary supplementation experiment.
