# Supplementary material for: Nicotinamide Riboside-Conditioned Microbiota Deflects High-Fat Diet-Induced Weight Gain in Mice
Source: mSystems. 2022 Jan 25;7(1):e00230-21. doi: 10.1128/msystems.00230-21 (PMC8788325; doi:10.1128/msystems.00230-21)
Supplement: TABLE S4 [file msystems.00230-21-st004.pdf]

41 KOs Enriched in NR-treated samples

KOs	Description
K10112	msmX, msmK, malK, sugC, ggtA, msiK; multiple sugar transport system ATP-binding protein
K10117	msmE; raffinose/stachyose/melibiose transport system substrate-binding protein
K10118	msmF; raffinose/stachyose/melibiose transport system permease protein
K10119	msmG; raffinose/stachyose/melibiose transport system permease protein
K02055	ABC.SP.S; putative spermidine/putrescine transport system substrate-binding protein
K20459	nukF, mcdF, sboF; lantibiotic transport system ATP-binding protein
K07718	yesM; two-component system, sensor histidine kinase YesM [EC:2.7.13.3]
K10439	rbsB; ribose transport system substrate-binding protein
K02052	ABC.SP.A; putative spermidine/putrescine transport system ATP-binding protein
K02057	ABC.SS.P; simple sugar transport system permease protein
K06596	chpA; chemosensory pili system protein ChpA (sensor histidine kinase/response regulator)
K11189	PTS-HPR; phosphocarrier protein
K01804	araA; L-arabinose isomerase [EC:5.3.1.4]
K20461	nukG, mcdG, sboG; lantibiotic transport system permease protein
K03315	nhaC; Na+:H+ antiporter, NhaC family
K03407	cheA; two-component system, chemotaxis family, sensor kinase CheA [EC:2.7.13.3]
K02405	fliA; RNA polymerase sigma factor for flagellar operon FliA
K02406	fliC; flagellin
K03412	cheB; two-component system, chemotaxis family, protein-glutamate methylesterase/glutaminase [EC:3.1.1.61 3.5.1.44]
K05813	ugpB; sn-glycerol 3-phosphate transport system substrate-binding protein
K03337	iolB; 5-deoxy-glucuronate isomerase [EC:5.3.1.30]
K23536	nupC; general nucleoside transport system permease protein
K10820	ytfR; galactofuranose transport system ATP-binding protein [EC:7.5.2.9]
K00008	SORD, gutB; L-iditol 2-dehydrogenase [EC:1.1.1.14]
K01996	livF; branched-chain amino acid transport system ATP-binding protein
K19157	yafQ; mRNA interferase YafQ [EC:3.1.-.-]
K00854	xylB, XYLB; xylulokinase [EC:2.7.1.17]
K07707	agrA, blpR, fsrA; two-component system, LytTR family, response regulator AgrA
K07706	agrC, blpH, fsrC; two-component system, LytTR family, sensor histidine kinase AgrC
K20491	nisE, spaE, cprB, epiE; lantibiotic transport system permease protein
K07240	chrA; chromate transporter
K02392	flgG; flagellar basal-body rod protein FlgG
K20487	nisK, spaK; two-component system, OmpR family, lantibiotic biosynthesis sensor histidine kinase NisK/SpaK [EC:2.7.13.3]
K07720	yesN; two-component system, response regulator YesN
K20488	nisR, spaR; two-component system, OmpR family, lantibiotic biosynthesis response regulator NisR/SpaR
K02556	motA; chemotaxis protein MotA
K00975	glgC; glucose-1-phosphate adenylyltransferase [EC:2.7.7.27]
K19309	bcrA; bacitracin transport system ATP-binding protein
K02004	ABC.CD.P; putative ABC transport system permease protein
K02003	ABC.CD.A; putative ABC transport system ATP-binding protein
K16786	ecfA1; energy-coupling factor transport system ATP-binding protein

49 KOs Enriched in Control-treated samples

KOs	Description
K02036	pstB; phosphate transport system ATP-binding protein [EC:7.3.2.1]
K02037	pstC; phosphate transport system permease protein
K03522	fixB, etfA; electron transfer flavoprotein alpha subunit
K01915	glnA, GLUL; glutamine synthetase [EC:6.3.1.2]
K02038	pstA; phosphate transport system permease protein
K23265	purQ; phosphoribosylformylglycinamidine synthase subunit PurQ / glutaminase
K01834	PGAM, gpmA; 2,3-bisphosphoglycerate-dependent phosphoglycerate mutase [EC:5.4.2.11]
K02040	pstS; phosphate transport system substrate-binding protein
K02073	metQ; D-methionine transport system substrate-binding protein
K01867	WARS, trpS; tryptophanyl-tRNA synthetase [EC:6.1.1.2]

K01869	LARS, leuS; leucyl-tRNA synthetase [EC:6.1.1.4]
K01689	ENO, eno; enolase [EC:4.2.1.11]
K02794	PTS-Man-EIIB, manX; PTS system, mannose-specific IIB component [EC:2.7.1.191]
K02069	ABC.X2.P; putative ABC transport system permease protein
K04567	KARS, lysS; lysyl-tRNA synthetase, class II [EC:6.1.1.6]
K03758	arcD, lysI, lysP; arginine:ornithine antiporter / lysine permease
K10823	oppF; oligopeptide transport system ATP-binding protein
K01870	IARS, ileS; isoleucyl-tRNA synthetase [EC:6.1.1.5]
K01873	VARS, valS; valyl-tRNA synthetase [EC:6.1.1.9]
K01874	MARS, metG; methionyl-tRNA synthetase [EC:6.1.1.10]
K01876	aspS; aspartyl-tRNA synthetase [EC:6.1.1.12]
K01879	glyS; glycyl-tRNA synthetase beta chain [EC:6.1.1.14]
K01878	glyQ; glycyl-tRNA synthetase alpha chain [EC:6.1.1.14]
K02435	gatC, GATC; aspartyl-tRNA(Asn)/glutamyl-tRNA(Gln) amidotransferase subunit C
K06158	ABCF3; ATP-binding cassette, subfamily F, member 3
K22132	tcdA; tRNA threonylcarbamoyladenosine dehydratase
K00865	glxK, garK; glycerate 2-kinase [EC:2.7.1.165]
K01425	glsA, GLS; glutaminase [EC:3.5.1.2]
K12573	rnr, vacB; ribonuclease R [EC:3.1.13.1]
K07667	kdpE; two-component system, OmpR family, KDP operon response regulator KdpE
K01883	CARS, cysS; cysteinyl-tRNA synthetase [EC:6.1.1.16]
K01889	FARSA, pheS; phenylalanyl-tRNA synthetase alpha chain [EC:6.1.1.20]
K02759	PTS-Cel-EIIA, celC, chbA; PTS system, cellobiose-specific IIA component
K00937	ppk; polyphosphate kinase [EC:2.7.4.1]
K02622	parE; topoisomerase IV subunit B [EC:5.6.2.2]
K07704	lytS; two-component system, LytTR family, sensor histidine kinase LytS [EC:2.7.13.3]
K15580	oppA, mppA; oligopeptide transport system substrate-binding protein
K15581	oppB; oligopeptide transport system permease protein
K15582	oppC; oligopeptide transport system permease protein
K15583	oppD; oligopeptide transport system ATP-binding protein
K01892	HARS, hisS; histidyl-tRNA synthetase [EC:6.1.1.21]
K02760	PTS-Cel-EIIB, celA, chbB; PTS system, cellobiose-specific IIB component
K00600	glyA, SHMT; glycine hydroxymethyltransferase [EC:2.1.2.1]
K00604	MTFMT, fmt; methionyl-tRNA formyltransferase [EC:2.1.2.9]
K07646	kdpD; two-component system, OmpR family, sensor histidine kinase KdpD [EC:2.7.13.3]
K01940	argG, ASS1; argininosuccinate synthase [EC:6.3.4.5]
K13038	coaBC, dfp; phosphopantothenoylcysteine decarboxylase/phosphopantothenate-cysteine ligase
K15771	ganP; arabinogalactan oligomer / maltooligosaccharide transport system permease protein
K08177	oxlT; MFS transporter, OFA family, oxalate/formate antiporter

Supplementary Table 4. Results of LEfSe analysis done on KOs found within the enriched pathways from Dietary supplementation experiment.
