# Supplementary material for: Nicotinamide Riboside-Conditioned Microbiota Deflects High-Fat Diet-Induced Weight Gain in Mice
Source: mSystems. 2022 Jan 25;7(1):e00230-21. doi: 10.1128/msystems.00230-21 (PMC8788325; doi:10.1128/msystems.00230-21)
Supplement: TABLE S5 [file msystems.00230-21-st005.pdf]

25 KOs Enriched in FMT-NR-treated samples

KOs	Description
K20509	madB, oadB, gcdB, mmdB; carboxybiotin decarboxylase [EC:7.2.4.1]
K20487	nisK, spaK; two-component system, OmpR family, lantibiotic biosynthesis sensor histidine kinase NisK/SpaK [EC:2.7.13.3]
K18350	vanSC, vanSE, vanSG; two-component system, OmpR family, sensor histidine kinase VanS
K18349	vanRC, vanRE, vanRG; two-component system, OmpR family, response regulator VanR
K18345	vanSB, vanS, vanSD; two-component system, OmpR family, sensor histidine kinase VanS
K17398	DNMT3A; DNA (cytosine-5)-methyltransferase 3A [EC:2.1.1.37]
K17319	lplB; putative aldouronate transport system permease protein
K04079	HSP90A, htpG; molecular chaperone HtpG
K03798	ftsH, hflB; cell division protease FtsH [EC:3.4.24.-]
K03581	recD; exodeoxyribonuclease V alpha subunit [EC:3.1.11.5]
K03563	csrA; carbon storage regulator
K03497	parB, spo0J; ParB family transcriptional regulator, chromosome partitioning protein
K03413	cheY; two-component system, chemotaxis family, chemotaxis protein CheY
K03205	virD4, lvhD4; type IV secretion system protein VirD4 [EC:7.4.2.8]
K02556	motA; chemotaxis protein MotA
K02417	fliNY, fliN; flagellar motor switch protein FliN/FliY
K02405	fliA; RNA polymerase sigma factor for flagellar operon FliA
K02035	ABC.PE.S; peptide/nickel transport system substrate-binding protein
K01998	livM; branched-chain amino acid transport system permease protein
K01995	livG; branched-chain amino acid transport system ATP-binding protein
K01972	E6.5.1.2, ligA, ligB; DNA ligase (NAD+) [EC:6.5.1.2]
K01835	pgm; phosphoglucomutase [EC:5.4.2.2]
K00688	PYG, glgP; glycogen phosphorylase [EC:2.4.1.1]

2 KOs Enriched in FMT-Control-treated samples

KOs	Description
K04958	ITPR1; inositol 1,4,5-triphosphate receptor type 1
K03655	recG; ATP-dependent DNA helicase RecG [EC:3.6.4.12]

Supplementary Table 5. Results of LEfSe analysis done on all KOs from the FMT experiment.
