# Supplementary material for: Nicotinamide Riboside-Conditioned Microbiota Deflects High-Fat Diet-Induced Weight Gain in Mice
Source: mSystems. 2022 Jan 25;7(1):e00230-21. doi: 10.1128/msystems.00230-21 (PMC8788325; doi:10.1128/msystems.00230-21)
Supplement: TABLE S6 [file msystems.00230-21-st006.pdf]

45 KOs Enriched in FMT-NR-treated samples

KOs	Description
K02315	dnaC; DNA replication protein DnaC
K01998	livM; branched-chain amino acid transport system permease protein
K01999	livK; branched-chain amino acid transport system substrate-binding protein
K01996	livF; branched-chain amino acid transport system ATP-binding protein
K01997	livH; branched-chain amino acid transport system permease protein
K01995	livG; branched-chain amino acid transport system ATP-binding protein
K13626	fliW; flagellar assembly factor FliW
K02035	ABC.PE.S; peptide/nickel transport system substrate-binding protein
K02421	fliR; flagellar biosynthetic protein FliR
K02420	fliQ; flagellar biosynthetic protein FliQ
K02419	fliP; flagellar biosynthetic protein FliP
K02412	fliI; flagellum-specific ATP synthase [EC:7.4.2.8]
K02410	fliG; flagellar motor switch protein FliG
K02416	fliM; flagellar motor switch protein FliM
K02417	fliNY, fliN; flagellar motor switch protein FliN/FliY
K02397	flgL; flagellar hook-associated protein 3 FlgL
K02390	flgE; flagellar hook protein FlgE
K02392	flgG; flagellar basal-body rod protein FlgG
K20487	nisK, spaK; two-component system, OmpR family, lantibiotic biosynthesis sensor histidine kinase NisK/SpaK [EC:2.7.13.3]
K03413	cheY; two-component system, chemotaxis family, chemotaxis protein CheY
K02557	motB; chemotaxis protein MotB
K02556	motA; chemotaxis protein MotA
K18350	vanSC, vanSE, vanSG; two-component system, OmpR family, sensor histidine kinase VanS
K03581	recD; exodeoxyribonuclease V alpha subunit [EC:3.1.11.5]
K22010	pdtaR; two-component system, response regulator PdtaR
K02401	flhB; flagellar biosynthetic protein FlhB
K02400	flhA; flagellar biosynthesis protein FlhA
K02405	fliA; RNA polymerase sigma factor for flagellar operon FliA
K02407	fliD; flagellar hook-associated protein 2
K18349	vanRC, vanRE, vanRG; two-component system, OmpR family, response regulator VanR
K18345	vanSB, vanS, vanSD; two-component system, OmpR family, sensor histidine kinase VanS [
K03412	cheB; two-component system, chemotaxis family, protein-glutamate methylesterase/glutaminase [EC:3.1.1.61 3.5.1.44]
K03415	cheV; two-component system, chemotaxis family, chemotaxis protein CheV
K02190	cbiK; sirohydrochlorin cobaltochelatase [EC:4.99.1.3]
K03205	virD4, lvhD4; type IV secretion system protein VirD4 [EC:7.4.2.8]
K18986	ihk; two-component system, OmpR family, sensor kinase Ihk [EC:2.7.13.3]
K00688	PYG, glgP; glycogen phosphorylase [EC:2.4.1.1]
K03666	hfq; host factor-I protein
K02226	cobC, phpB; alpha-ribazole phosphatase [EC:3.1.3.73]
K17398	DNMT3A; DNA (cytosine-5)-methyltransferase 3A [EC:2.1.1.37]
K07707	agrA, blpR, fsrA; two-component system, LytTR family, response regulator AgrA
K02469	gyrA; DNA gyrase subunit A [EC:5.6.2.2]
K03563	csrA; carbon storage regulator
K02388	flgC; flagellar basal-body rod protein FlgC
K20490	nisF, spaF, cprA, epiF; lantibiotic transport system ATP-binding protein

28 KOs Enriched in FMT-Control-treated samples

KOs	Description
K01104	E3.1.3.48; protein-tyrosine phosphatase [EC:3.1.3.48]
K00426	cydB; cytochrome bd ubiquinol oxidase subunit II [EC:7.1.1.7]
K03272	gmhC, hldE, waaE, rfaE; D-beta-D-heptose 7-phosphate kinase / D-beta-D-heptose 1-phosphate adenosyltransferase [EC:2.7.1.167 2.7.7.70]
K02899	RP-L27, MRPL27, rpmA; large subunit ribosomal protein L27
K02892	RP-L23, MRPL23, rplW; large subunit ribosomal protein L23
K02890	RP-L22, MRPL22, rplV; large subunit ribosomal protein L22

K02931	RP-L5, MRPL5, rplE; large subunit ribosomal protein L5
K01187	malZ; alpha-glucosidase [EC:3.2.1.20]
K03551	ruvB; holliday junction DNA helicase RuvB [EC:3.6.4.12]
K02982	RP-S3, rpsC; small subunit ribosomal protein S3
K03086	rpoD; RNA polymerase primary sigma factor
K02078	acpP; acyl carrier protein
K02946	RP-S10, MRPS10, rpsJ; small subunit ribosomal protein S10
K02886	RP-L2, MRPL2, rplB; large subunit ribosomal protein L2
K02904	RP-L29, rpmC; large subunit ribosomal protein L29
K02906	RP-L3, MRPL3, rplC; large subunit ribosomal protein L3
K02621	parC; topoisomerase IV subunit A [EC:5.6.2.2]
K02992	RP-S7, MRPS7, rpsG; small subunit ribosomal protein S7
K02472	wecC; UDP-N-acetyl-D-mannosaminuronic acid dehydrogenase [EC:1.1.1.336]
K02874	RP-L14, MRPL14, rplN; large subunit ribosomal protein L14
K03628	rho; transcription termination factor Rho
K03655	recG; ATP-dependent DNA helicase RecG [EC:3.6.4.12]
K00971	manC, cpsB; mannose-1-phosphate guanylyltransferase [EC:2.7.7.13]
K01809	manA, MPI; mannose-6-phosphate isomerase [EC:5.3.1.8]
K02913	RP-L33, MRPL33, rpmG; large subunit ribosomal protein L33
K07636	phoR; two-component system, OmpR family, phosphate regulon sensor histidine kinase PhoR
K02965	RP-S19, rpsS; small subunit ribosomal protein S19
K02864	RP-L10, MRPL10, rplJ; large subunit ribosomal protein L10

Supplementary Table 6. Results of LEfSe analysis done on KOs found within the enriched pathways from the FMT experiment.
